# *Porphyromonas gingivalis* outer membrane vesicles increase vascular permeability by inducing stress fiber formation and degrading vascular endothelial‐cadherin in endothelial cells

**DOI:** 10.1111/febs.17349

**Published:** 2024-12-17

**Authors:** Mana Mekata, Kaya Yoshida, Ayu Takai, Yuka Hiroshima, Ayu Ikuta, Mariko Seyama, Kayo Yoshida, Kazumi Ozaki

**Affiliations:** ^1^ Department of Oral Healthcare Promotion, Graduate School of Biomedical Sciences Tokushima University Japan; ^2^ Department of Oral Microbiology, Graduate School of Biomedical Sciences Tokushima University Japan

**Keywords:** outer membrane vesicles, periodontal diseases, *Porphyromonas gingivalis*, vascular permeability

## Abstract

*Porphyromonas gingivalis* (*Pg*) is a keystone bacterium associated with systemic diseases, such as diabetes mellitus and Alzheimer's disease. Outer membrane vesicles (OMVs) released from *Pg* have been implicated in systemic diseases by delivering *Pg* virulence factors to host cells in distant organs and inducing cellular dysfunction. *Pg* OMVs also have the potential to enter distant organs via the bloodstream. However, the effects of *Pg* OMVs on the vascular function are poorly understood. Here, we showed that *Pg* OMVs increase vascular permeability by promoting stress fiber formation and lysosome/endosome‐mediated vascular endothelial‐cadherin (VEc) degradation in human umbilical vein endothelial cells (HUVECs) and human pulmonary microvascular endothelial cells (HPMECs). F‐actin, visualized via fluorescein isothiocyanate‐phalloidin, became thicker and longer, leading to the formation of radical stress fibers in response to *Pg* OMVs in HUVECs and HPMECs. Western blotting and quantitative real‐time polymerase chain reaction analyses revealed that *Pg* OMVs decreased VEc protein levels in a gene‐independent manner. *Pg* OMVs enhanced vesicular VEc accumulation in the cytoplasm around lysosome‐associated membrane protein 1‐positive structures during pretreatment with the lysosomal inhibitor chloroquine. This suggests that *Pg* OMVs decrease VEc protein levels by accelerating their internalization and degradation via lysosomes and endosomes. A27632 inhibition of Rho kinases impaired the *Pg* OMV‐induced stress fiber formation and VEc degradation, resulting in the recovery of hyperpermeability. These findings provide new insights into the pathogenesis of systemic diseases that are associated with periodontal diseases.

## Graphical abstract

*Porphyromonas gingivalis* (*Pg*), a periodontal bacterium implicated in systemic diseases, release outer membrane vesicles (OMVs). Here, we show that *Pg* OMVs induce stress fiber formation and degrade vascular endothelial cadherin (VEc) via lysosomes and endosomes in endothelial cells, leading to hyper‐permeability. The inhibition of Rho kinases impairs this. These findings provide new insights into the pathogenesis of systemic diseases associated with periodontal diseases.

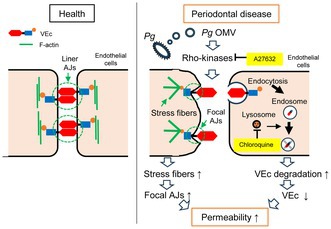

**Abbreviations**
AJs: adhesion junctions
BSA: bovine serum albumin
FITC: fluorescein isothiocyanate
HPMECs: human pulmonary microvascular endothelial cells
HUVECs: human umbilical vein endothelial cells
LAMP‐1: lysosome‐associated membrane protein 1
LPS: lipopolysaccharide
OMVs: outer membrane vesicles
PBS: phosphate‐ buffered saline
PCAM‐1: platelet and endothelial adhesion molecule‐1
*Pg*
: *Porphyromonas gingivalis*
PMSF: phenylmethylsulphonyl fluoride
RhoA: Ras homolog gene family member A
SDS/‐PAGE: sodium dodecyl sulfate‐polyacrylamide gel electrophoresis
TNF‐α: tumor necrosis factor‐α
VEc: vascular endothelial‐cadherin
VEGF: vascular endothelial growth factor

## Introduction

*Porphyromonas gingivalis* (*Pg*) is an oral pathogen associated with chronic periodontitis. It is associated with many systemic diseases, including diabetes mellitus, cardiovascular disease, and Alzheimer's disease [1, 2, 3, 4]. *Pg* produces outer membrane vesicles (OMVs), which shed from its outer membrane, have a diameter of 100–150 nm and are loaded with the majority of *Pg* virulence factors such as gingipains, fimbriae, and lipopolysaccharide (LPS) [5]. *Pg* OMVs released into the extracellular space can adhere to and invade host cells, leading to cellular dysfunction [6, 7]. It has been hypothesized that periodontal disease‐related systemic diseases may be mediated by mechanisms whereby *Pg* OMVs deliver *Pg* virulence factors to host cells in distant organs [8, 9].

The relationship between *Pg* OMVs and systemic diseases was evaluated in mouse models. Middle‐aged mice challenged with *Pg* OMVs through oral gavage showed OMV localization in the hippocampus and cortex, causing memory dysfunction and neuroinflammation [10]. In previous studies, we observed that *Pg* OMVs injected into the abdominal cavity of mice translocated to the brain and liver, leading to neuroinflammation and diabetes mellitus [11, 12]. These findings suggest that *Pg* OMVs can enter the bloodstream, reach distant organs, and cause leakage that affects cellular function. However, it is unclear how *Pg* OMVs affect vascular function by moving into vessels and leaking outside into distant tissues and organs.

Endothelial cells lining the vessel wall form an endothelial barrier to separate blood from peripheral tissues and control vascular permeability to maintain tissue–fluid homeostasis via paracellular pathways [13]. Vascular permeability increases under several pathological conditions, including inflammation, and leads to the efflux of fluid and circulating cells, resulting in edema and hemorrhage [14]. Endothelial barrier leakage (hyperpermeability) is mainly mediated by two mechanisms. One is the downregulation of protein complexes composed of endothelial adhesion junctions (AJs) and tight junctions, such as vascular endothelial‐cadherin (VEc), claudin 5, and platelet and endothelial adhesion molecule‐1 (PCAM‐1) [15]. VEc is specifically expressed in endothelial cells and is a major adhesion molecule in endothelial AJs, regulating vascular permeability by binding to the actin cytoskeleton through several proteins such as p120‐catenin and β‐catenin in the cytoplasmic domain [16]. Several studies have shown that VEc reduction in endothelial cells can induce hyperpermeability *in vivo* and *in vitro* [17]. Furthermore, the loss of VEc is mediated by its internalization through endocytosis, followed by its degradation via the lysosome/endosome pathway in response to the dissociation of p120 from the VEc cytoplasmic domain [18, 19].

The reorganization of actin cytoskeletons anchored to VEc‐based‐AJs also increases vascular permeability [20]. AJs usually exist along cell–cell junctions and are supported by circumferential actin bundles. Therefore, they are depicted with a linear morphology, called linear AJs. In linear AJs, VEc is stabilized by connecting circumferential actin bundles through α‐ and β‐catenin; therefore, the endothelial barrier is strong with normal vascular permeability [21]. In contrast, inflammatory mediators induce discontinuous focal AJs connected to thick radial actin bundles (stress fibers) anchored to the VEc on focal AJs under pathological conditions such as chronic inflammation [22, 23]. These stress fibers generate a pulling force on focal AJs and reduce the endothelial barrier, resulting in hyperpermeability [24].

There is little evidence that periodontal diseases participate in the formation of stress fibers and focal AJs in the endothelium. By contrast, periodontal bacteria and their products reportedly increase vascular permeability by reducing the protein levels of adhesion molecules. Farrugia *et al*. reported that *Pg* infections damaged zebrafish larval microvessels and increased vascular permeability by degrading PCAM‐1 and VEc via the *Pg*‐specific protease gingipains [25]. *Pg* OMVs promote vascular permeability by inducing cell death in retinal vascular endothelial cells or cerebral microvascular endothelial cells by degrading zonula occludin‐1 and occludin [26, 27]. These findings suggest that periodontal disease may be implicated in hyperpermeability in various systemic diseases, although the underlying molecular mechanisms are not well‐known.

This study investigated how *Pg* OMVs mediate vascular permeability in endothelial cells. *Pg* OMVs induced stress fiber formation and lysosome/endosome‐mediated VEc degradation, which were dependent on Rho kinases. These findings suggest that *Pg* OMVs may contribute to the pathogenesis of systemic diseases by affecting vascular permeability.

## Results

### *Pg* OMVs increase vascular permeability *in vivo* and *in vitro*

**Fig. 1:**
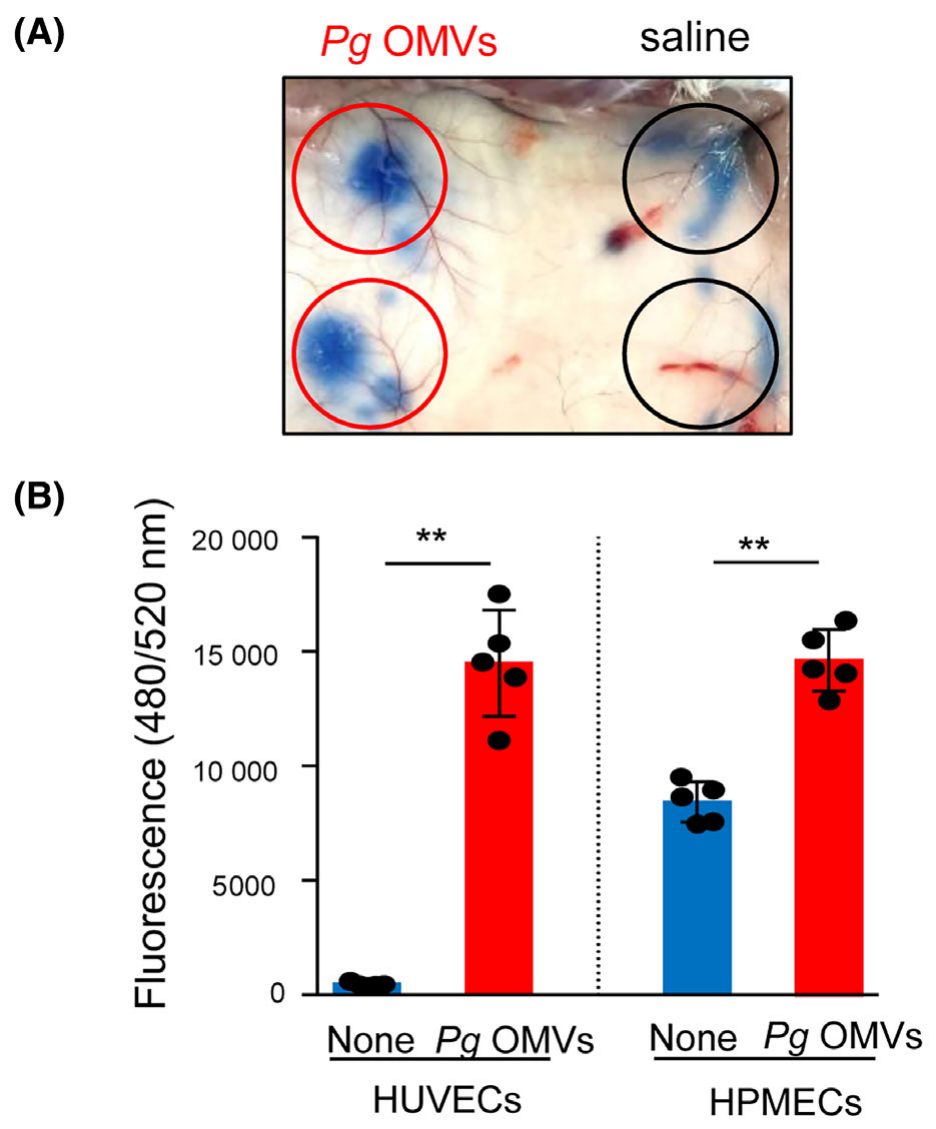
*Pg* OMVs increase vascular permeability *in vivo* and *in vitro*. (A) Representative dorsal skin images of mice in the Miles assay. *Porphyromonas gingivalis* (*Pg*) outer membrane vesicles (OMVs) containing 15 μg of protein (right side of the skin) or saline (left side of the skin) were singly injected into dorsal skin of BALB/cAJc1 mice. Subsequently, 100 μL of 0.5% Evans blue was injected into the tail vein. After 10 min, the gross Evans blue dye level was visually observed on the skin surface in *Pg* OMVs‐injected area (red circles) and saline‐injected area (black circles). Four mice were used per group for each experiment and a typical image of visual discoloration is shown. (B) Human umbilical vein endothelial cells (HUVECs) and human pulmonary microvascular endothelial cells (HPMECs) were grown to confluence on the insert of a transwell system and treated with (*n* = 5) or without (none, *n* = 4) *Pg* OMVs (500 ng·mL^−1^) for 60 min. FITC‐labeled dextran (1 mg·mL^−1^) was then added to the insert. After 10 min, the FITC‐labeled dextran leakage from the apical compartment of the insert to the bottom well was quantified by measuring the fluorescence intensity. Student's *t*‐test was used for statistical analysis. Error bars represent standard deviation. **P < 0.01 compared with no‐treated cells (None).

The Miles assay was used to detect microvascular leakage and assess vascular permeability *in vivo* [28]. Evans blue is a dye that noncovalently binds to albumin and is injected intravenously as a tracer. The gross Evans blue dye level, reflecting the leakage of the dye‐albumin complex from the vascular compartment, was visually observed on the mouse skin surface. Saline was injected into the skin on the left dorsal side of the mice as a control, whereas 15 μg of *Pg* OMVs was injected into the skin on the dorsal right side. The injection of *Pg* OMVs into the mouse skin increased Evans blue exudation 10 min after injection compared to the control (Fig. 1A).

Subsequently, HUVECs or HPMECs were cultured to confluence on the inserts of the transwell system and treated with 500 ng·mL^−1^ *Pg* OMVs for 60 min. Permeability was evaluated by measuring the fluorescence of 70 kDa FITC‐dextran passing through the monolayer to the bottom wells. Fluorescence significantly increased after *Pg* OMV challenge in HUVECs and HPMECs (Fig. 1B). This indicates that *Pg* OMVs increased vascular permeability in endothelial cells *in vitro*.

### *Pg* OMVs induce stress fiber formation in HUVECs and HPMECs

**Fig. 2:**
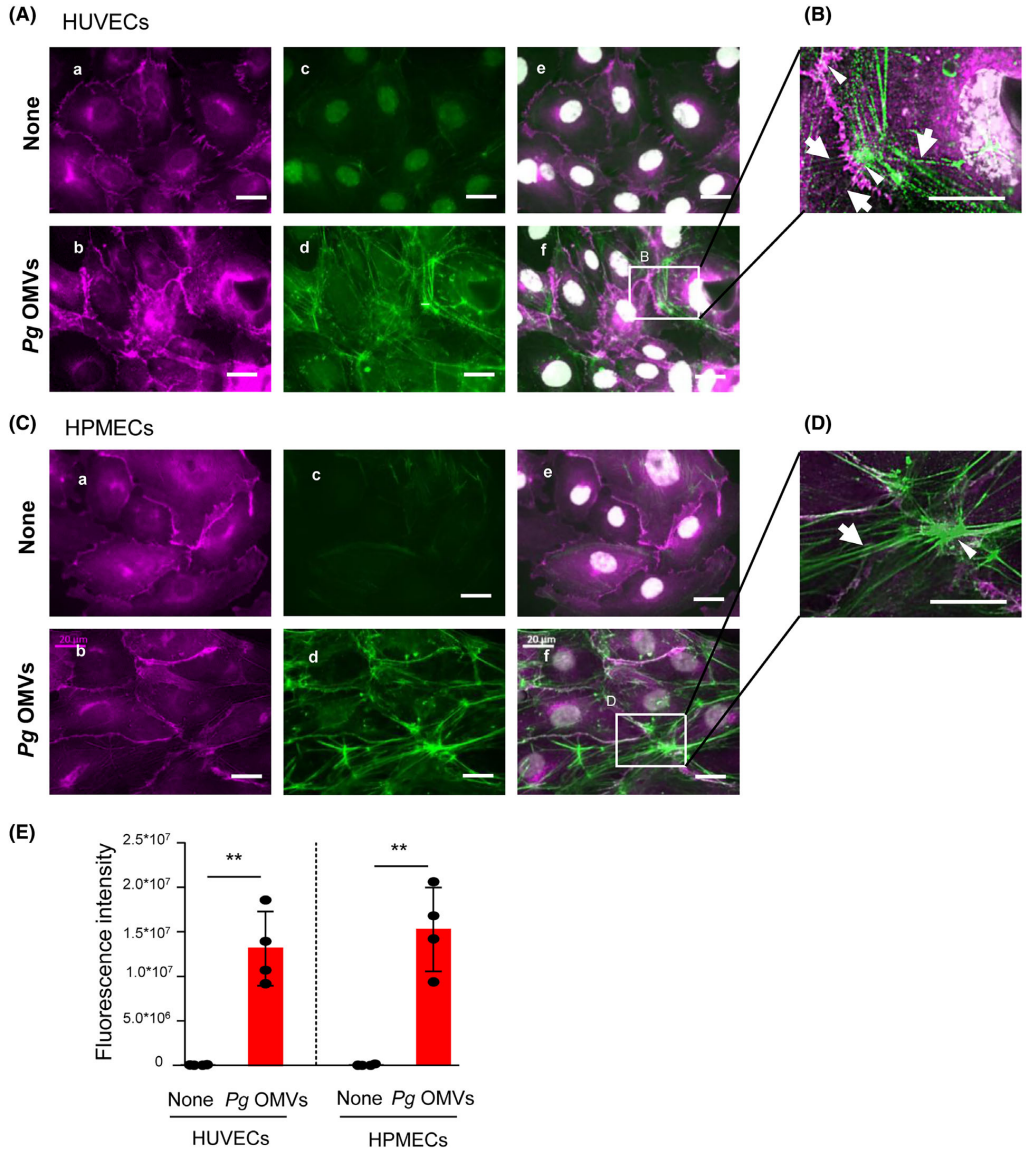
*Pg* OMVs induce stress fiber formation in HUVECs and HPMECs. HUVECs (A, B) and HPMECs (C, D) were cultured in monolayers and treated with (B, D, F) or without (none; A, C, E) 500 ng·mL^−1^ of *Porphyromonas gingivalis* (*Pg*) outer membrane vesicles (OMVs) for 60 min. Cells were fixed and stained with vascular endothelial‐cadherin (VEc) to label adhesion junctions (AJs) (magenta) (A, B). F‐actin was labeled with FITC‐phalloidin (green) (C, D), and the nuclei were stained with Hoechst 33342 (white) and merged (E, F). High‐magnification merged images (2.5×) are shown in (B) and (D). Arrows indicate radical stress fibers. The arrowhead represents focal AJs connected to stress fibers. Scale bars indicate 20 μm. *n* = 4 in each group. Four samples were set up in each group (*n* = 4), and the typical photographs were shown from four independent experiments. (E). F‐actin was labeled with FITC‐phalloidin (green) in HUVECS and HPMECs treated with or without 500 ng·mL^−1^ of *Pg* OMVs for 60 min (*n* = 4 in each group), and green fluorescence intensity were measured. Student's *t*‐test was used for statistical analysis. Error bars represent standard deviation. **P < 0.01 compared with no‐treated cells (None).

Stress fiber formation is the major mechanism that reduces endothelial barrier function and induces hyperpermeability [21]. Therefore, we examined whether *Pg* OMVs challenge induced changes in the actin cytoskeleton of endothelial cells. Immunofluorescence was used label AJs via staining of VEc, and F‐actin was visualized using FITC‐phalloidin. Untreated HUVECs exhibited minimal assembly of peripheral F‐actin along the cell margins and were very thin (Fig. 2A,C,E). In contrast, *Pg* OMVs challenge increased the thickness and length of F‐actin (Fig. 2A,D,F). *Pg* OMVs‐treated HUVECs exhibited radical stress fibers (Fig. 2B, arrows), with connected focal AJs (Fig. 2B, arrowheads). Similarly, *Pg* OMVs‐treated HPMECs showed a remarkable increase in thick and elongated F‐actin bundles (Fig. 2C,D,F), radial stress fibers (Fig. 2D, arrows), and focal AJs (Fig. 2D, arrowheads). The fluorescence intensity of F‐actin in the images of HUVECs and HPMECs treated with or without *Pg* OMVs was quantified. As shown in Fig. 2E, *Pg* OMVs significantly increased stress fiber formation in both HUVECs (*P* = 0.0008) and HPMECs (*P* = 0.0007) (Fig. 2E). These results showed that *Pg* OMVs induced F‐actin reorganization into stress fibers in endothelial cells.

### *Pg* OMVs downregulate the level of VEc protein but not mRNA in HUVECs and HPMECs

**Fig. 3:**
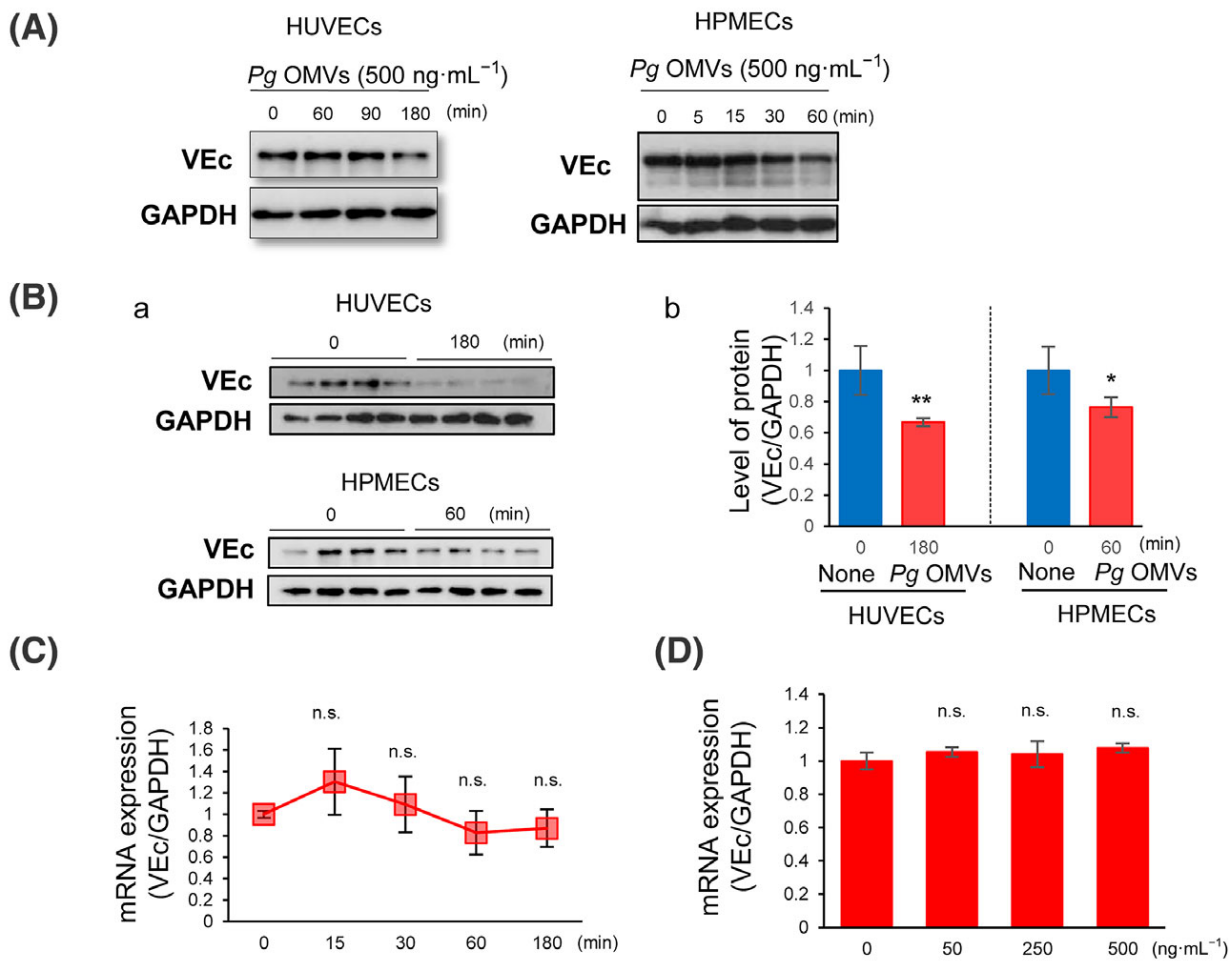
*Pg* OMVs downregulate the level of VEc protein but not mRNA in HUVECs and HPMECs. (A) Human umbilical vein endothelial cells (HUVECs) and human pulmonary microvascular endothelial cells (HPMECs) were treated with 500 ng·mL^−1^ *Porphyromonas gingivalis* (*Pg*) outer membrane vesicles (OMVs). Subsequently, vascular endothelial‐cadherin (VEc) expression was analyzed via western blotting at indicated periods. (B) HUVECs and HPMECs were treated with *Pg* OMVs for 0 or 180 min, and 60 min, respectively. (A) The cell lysates were analyzed via western blotting using antibodies for VEc and GAPDH. (B) Densitometric analysis of each band was performed using imagej, and the VEc/GAPDH ratio in each group were presented and analyzed using Student's *t*‐test (*n* = 4 in each group). Values are shown as the fold change in expression levels compared to that in the 0 min group. Error bars represent standard deviation. *P < 0.05, **P < 0.01 compared with 0 min. (C) HUVECs were treated with 500 ng·mL^−1^ *Pg* OMVs for 0–180 min (*n* = 4). (D) HUVECs were treated with 0–500 ng·mL^−1^ *Pg* OMVs for 180 min (*n* = 4). VEc mRNA expression was analyzed using real‐time PCR. Values are shown as the fold change in expression levels compared to that in the 0 min group. Student's *t*‐test was used for statistical analysis (*n* = 4 in each group; n.s., not significant). Error bars represent standard deviation.

Downregulation of VEc in AJs is critical for increased vascular permeability [15]. Therefore, we analyzed the expression levels of the VEc protein in *Pg* OMVs‐treated endothelial cells using western blotting. HUVECs challenged with *Pg* OMV showed decreased VEc expression after 180 min of incubation. HPMECs challenged with *Pg* OMVs showed decreased VEc protein levels after 60 min of incubation (Fig. 3A). Quantitative analysis of HUVECs and HPMECs treated with *Pg* OMVs for 180 or 60 min was performed by measuring VEc protein levels via western blotting (Fig. 3B‐a, upper panel). The densities of bands for VEc and GAPDH were quantified using imagej software, and VEc protein levels in each group were statistically analyzed using Student's *t*‐test (Fig. 3B‐b). The VEc protein level was significantly decreased at 180 min and 60 min after *Pg* OMVs challenge compared to that at 0 min in HUVECs (*P* = 0.004) and HPMECs (*P* = 0.032), respectively.

Meanwhile, *the Pg* OMVs challenge did not affect VEc mRNA expression at 0–180 min (Fig. 3C). To confirm this result, HUVECs were treated with various concentrations of *Pg* OMVs (0–500 ng·mL^−1^) for 180 min. We found that treatment with *Pg* OMVs did not alter VEc mRNA expression (Fig. 3D). This indicates that *Pg* OMVs regulate VEc protein levels by other mechanisms besides decreasing mRNA levels.

### *Pg* OMVs accelerate VEc degradation through the lysosomal pathway in HUVECs

**Fig. 4:**
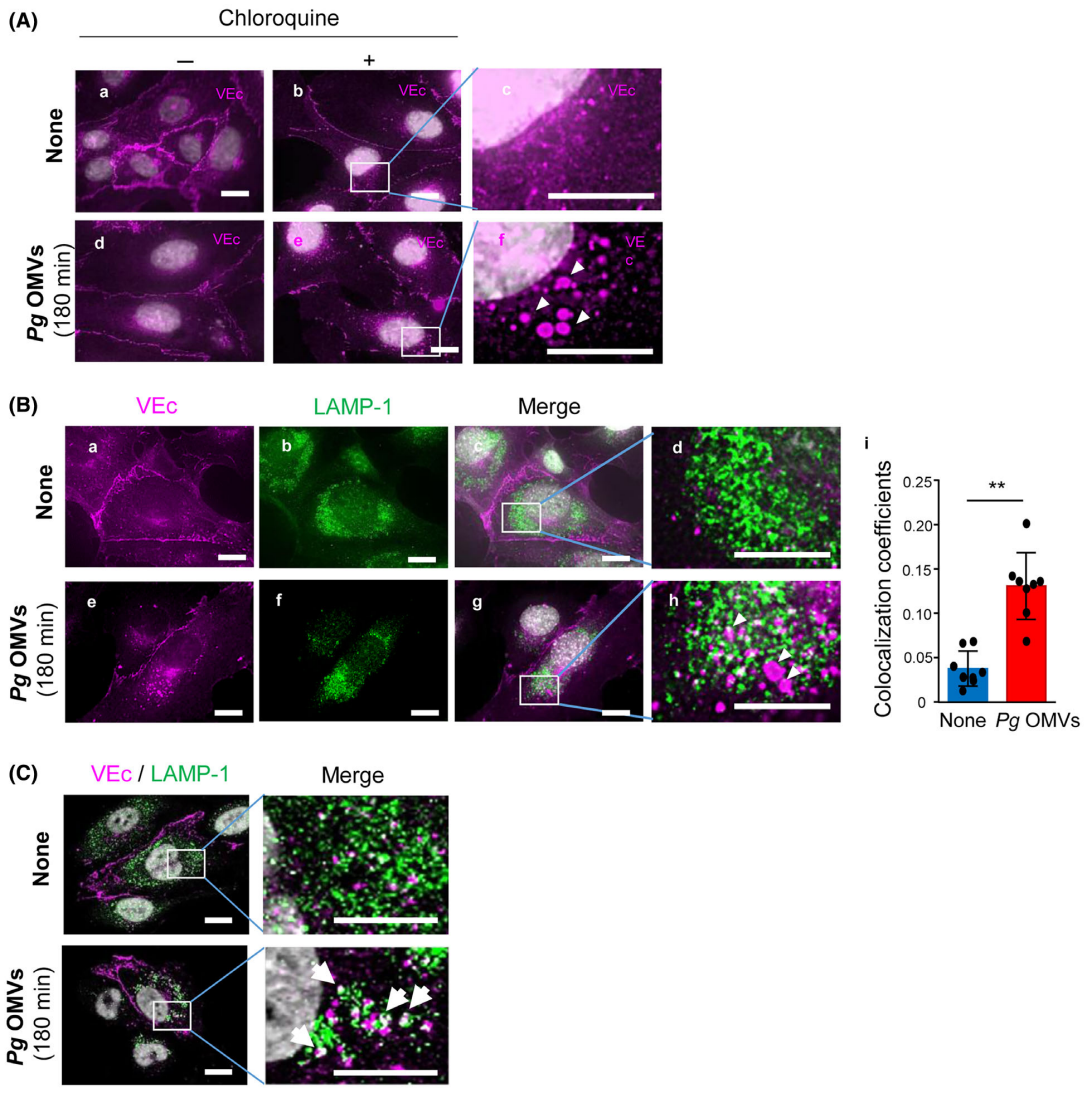
*Pg* OMVs accelerate VEc degradation through the lysosomal pathway in HUVECs. (A) Human umbilical vein endothelial cells (HUVECs) were pretreated with (+) (B, C, E, F) or without (−) (A, D) chloroquine for 30 min. Subsequently, cells were treated with (D–F) or without (A–C) 500 ng·mL^−1^ *Porphyromonas gingivalis* (*Pg*) outer membrane vesicles (OMVs) for 180 min before fixation and subjected to vascular endothelial‐cadherin (VEc) staining for immunofluorescence analysis. Nuclei were stained with Hoechst 33342. High‐magnification images (4×) of (B) and (E) are shown (C, F). Scale bar, 20 μm. Arrowheads indicate the accumulation of intracellular vesicular VEc. Four samples were set up in each group (*n* = 4), and the typical photographs were shown from three independent experiments. (B) Vesicular VEc was induced by same method shown as (A). VEc and lysosome‐associated membrane protein 1 (LAMP‐1) were co‐stained with specific antibodies for VEc (A, E) or LAMP‐1 (B, F). Nuclei were stained with Hoechst 33342 and the microscopic images of the same field were merged (C, G). High‐magnification images (4×) of the merge are shown (D, H). Scale bars indicate 20 μm. Arrowheads indicate vesicular VEc surrounded by LAMP‐1. (I) The merged white fluorescent intensity and green fluorescent intensity around nucleus were quantified. The ratio of merged (white)/LAMP‐1 (green) is shown as the co‐localization coefficients of VEc and LAMP‐1. Student's *t*‐test was used for statistical analysis (*n* = 4, samples; *n* = 8, field of view, in each group). Error bars represent standard deviation. **P < 0.01 compared with no‐treated cells (None). (C) The same samples used in (B) were subjected to confocal microscopy (A, C), and high‐magnification images (4×) are shown in (B, D). VEc and LAMP‐1 double‐staining revealed co‐localization in some vesicles induced by *Pg* OMVs (D, arrows).

Endothelial permeability may be regulated by VEc internalization via clathrin‐dependent endocytosis [19]. The internalized VEc is degraded through the lysosomal/endosomal pathway [18]. HUVECs were treated with chloroquine (a lysosome inhibitor that impairs lysosomal acidification [29]) to determine whether the *Pg* OMVs‐induced reduction in VEc protein levels resulted from dysfunction of the lysosomal/endosomal pathway. VEc staining was observed in control cells as cell surface clusters (Fig. 4A‐a), and no punctate staining for VEC was detected in the cytoplasm after chloroquine treatment (Fig. 4A–C). VEc was primarily observed around the cell margin in *Pg* OMVs‐treated HUVECs, which was the same as that in control cells (Fig. 4A,D); however, chloroquine dramatically increased punctate VEc staining in the cytoplasm (Fig. 4A,E,F; arrowheads). These results suggest that *Pg* OMVs can induce VEc internalization via endocytosis and localization to cytoplasmic vesicles when lysosomal protein degradation is inhibited by chloroquine.

We performed co‐immunofluorescence for VEc and lysosome‐associated membrane protein 1 (LAMP‐1, a known marker of lysosomes and late endosomes) to provide further evidence that vesicular VEc is processed through the lysosomal and endosomal pathways [30] in *Pg* OMVs‐treated HUVECs. *Pg* OMVs induced the accumulation of vesicular VEc in the cytoplasm in the presence of chloroquine (Fig. 4B,E), in contrast with control cells where most VEc was observed in the cell margin (Fig. 4B‐a). Positive staining for LAMP‐1 was observed around the nucleus of HUVECs treated with or without *Pg* OMVs (Fig. 4B‐b,F). Most VEc‐positive vesicles in *Pg* OMVs‐treated HUVECs were localized around LAMP‐1‐positive vesicular structures (Fig. 4B,G,H, arrowheads) compared to control cells (Fig. 4B–D). The merged yellow and green fluorescence intensities were quantified, and the ratio of merged (yellow)/LAMP‐1 (green) was calculated as the co‐localization coefficient (Fig. 4B,I). *Pg* OMVs significantly increased co‐localization coefficients (*P* = 2.38 × 10^−5^).

Confocal microscopy further confirmed increased vesicular VEc accumulation in the cytoplasm of *Pg* OMVs‐treated cells in the presence of chloroquine, compared to that in control cells, similar to the results shown in Fig. 4B. Notably, some VEc‐positive vesicles co‐localized with the LAMP‐1‐positive structure (Fig. 4C,D, arrows), suggesting that a part of the VEc was finally directed to and contained in the lysosome/late endosome in response to *Pg* OMVs.

Taken together, we conclude that *Pg* OMVs can accelerate VEc protein internalization and degradation through lysosomal and endosomal pathways in HUVECs.

### Rho kinases are involved in *Pg* OMVs‐induced permeability in HUVECs

**Fig. 5:**
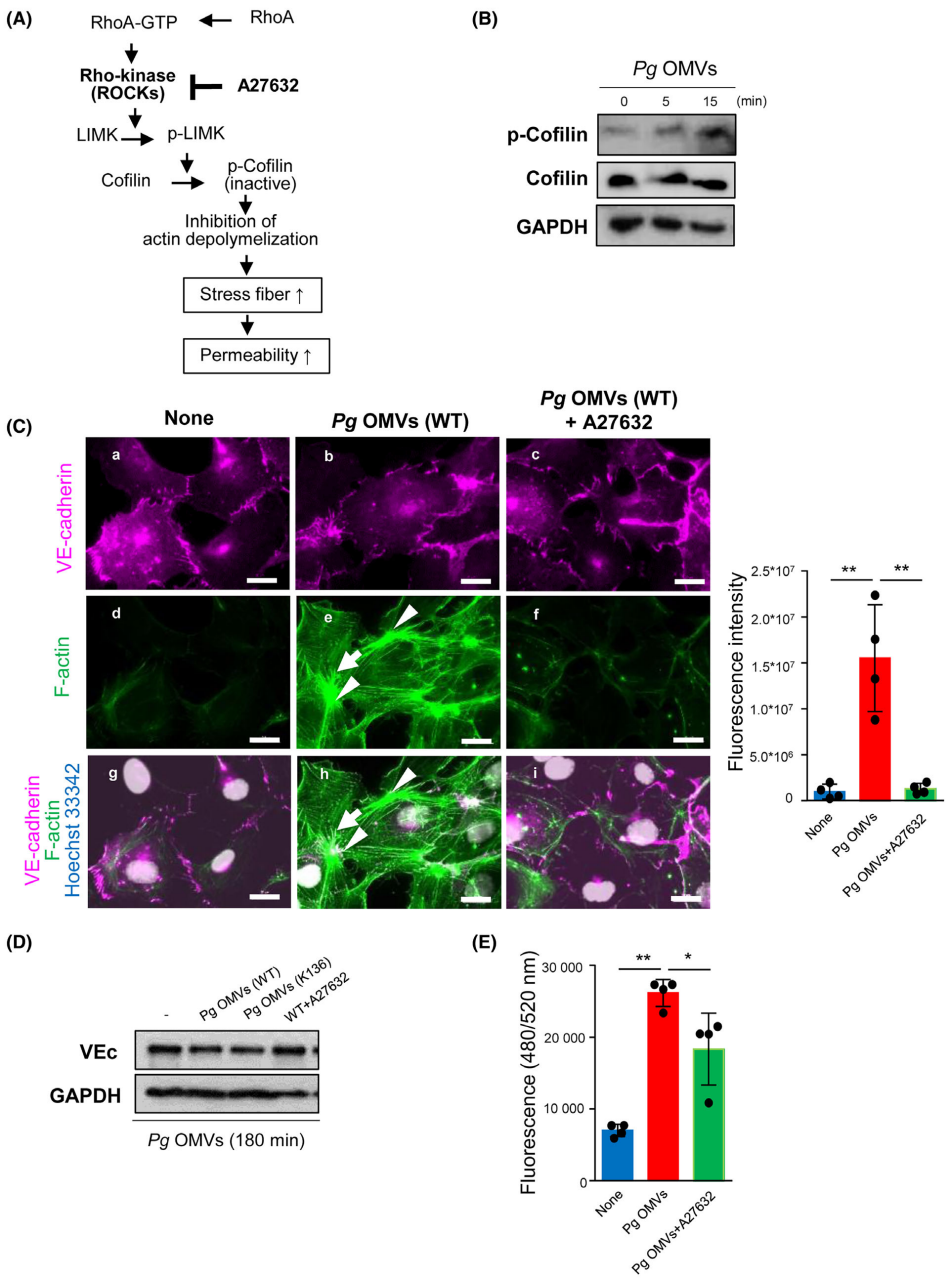
Rho kinases are involved in *Pg* OMVs‐induced permeability in HUVECs. (A) The hyperpermeability signaling pathway promoted by actin filament rearrangement. (B) Human umbilical vein endothelial cells (HUVECs) were treated with 500 ng·mL^−1^ *Porphyromonas gingivalis* (*Pg*) outer membrane vesicles (OMVs) for the indicated periods and cofilin phosphorylation was assessed via western blotting. The immunoblots are representative figures of three independent experiments. (C) HUVECs were pretreated with 10 μm Y27632 for 30 min before *Pg* OMVs challenge for 60 min (C, F, I). Cells were treated, fixed, and stained as described in Fig. 2. Arrows indicate the radical stress fibers (E, H). The arrowhead indicates focal adhesion junctions (AJs) connected to stress fibers (E, H). Bars indicate 20 μm. The fluorescence intensity of F‐actin labeled with FITC‐phalloidin (green, in D, E and F) was quantified (J). Student's *t*‐test was used for statistical analysis (*n* = 4 in each group). Error bars represent standard deviation. **P < 0.01 compared with no‐treated cells (None). (D) HUVECs were pretreated with 10 μm Y27632 for 30 min before *Pg* OMVs challenge for 180 min. Vascular endothelial‐cadherin (VEc) protein levels were analyzed via western blotting. WT, wild‐type; KDP136, gingipain deficient. (E) HUVECs were grown on Transwell inserts and pretreated with 10 μm Y27632 for 30 min before *Pg* OMVs challenge for 60 min. Vascular permeability was assessed as shown in Fig. 1B. Student's *t*‐test was used for statistical analysis (*n* = 4). Error bars represent standard deviation. **P < 0.01.

A known signaling pathway that increases endothelial permeability promoted by the reorganization of actin filaments is shown in Fig. 5A [31]. Briefly, a member of small GTPases called Ras homolog gene family member A (RhoA) is activated by permeability‐inducing factors such as vascular endothelial growth factor (VEGF) and tumor necrosis factor‐α (TNF‐α). Rho‐A then binds to and activates downstream Rho kinases (also known as Rho‐associated coiled‐coil containing protein kinases). Activated Rho kinases phosphorylate LIM kinase, resulting in cofilin phosphorylation. Cofilin inactivation by phosphorylation inhibits actin depolymerization, stabilizes actin filaments, and enhances stress fiber formation (Fig. 5A).

*Pg* OMVs induced cofilin phosphorylation in HUVECs 15 min after treatment, suggesting that *Pg* OMVs activate the actin rearrangement pathway (Fig. 5B). We further examined whether Rho kinases could regulate endothelial permeability in *Pg* OMV‐treated HUVECs using Y27632, a Rho kinase inhibitor. Y27632 attenuated *Pg* OMVs‐induced radial stress fibers (Fig. 5C, arrows) and focal AJs in HUVECs (Fig. 5C, arrowheads). Inhibition of Rho kinases by Y27632 also restored VEc protein levels, which were reduced by treatment with *Pg* OMVs for 180 min (Fig. 5D). In addition, Y27632 partially attenuated *Pg* OMVs‐induced hyperpermeability in HUVECs (Fig. 5E).

### *Pg* OMVs induced increase in permeability is unaffected by gingipains

*Pg*‐specific proteases including gingipains Kgp and Rgp, which are abundant in *Pg* cells and OMVs, promote vascular permeability by degrading adhesion proteins [25, 32]. Therefore, we investigated whether gingipains are implicated in *Pg* OMVs‐induced stress fiber formation or VEc degradation. OMVs were isolated from gingipain‐deficient *Pg* (KDP136) culture media [11]. We compared its effects to those of *Pg* OMVs (WT) isolated from *Pg* which normally expresses gingipains.

First, we examined whether the gingipains contained in *Pg* OMVs could degrade VEc proteins. Endogenous VEc proteins were immunoprecipitated from HUVECs using antibodies for VEc. The precipitated VEc was incubated with *Pg* OMVs derived from *Pg* (WT) or *Pg* (KDP136) for 30 min and then subjected to SDS/PAGE, and VEc protein levels were analyzed using western blotting. Immunoprecipitated VEc markedly decreased after incubation with *Pg* OMVs (WT) or with the culture media of *Pg* (WT) (*Pg* (WT) sup), which is abundant in secreted gingipains (Fig. S1A). In contrast, gingipain‐deficient *Pg* OMVs (KDP136) did not affect VEc levels. This suggests that gingipains in *Pg* OMVs possess protease activity and can proteolytically digest VEc (Fig. S1A).

In contrast, *Pg* OMVs (KDP136) decreased VEc protein levels to the same degree as *Pg* OMVs (WT) in HUVECs (Fig. 5D). *Pg* OMVs (KDP136) also increased stress fiber formation and AJs in HUVECs (Fig. S1B‐f,i). The stress fiber formation was quantified and compared in *Pg* OMVs (WT) and *Pg* OMVs (KDP136)‐treated HUVECs. *Pg* OMVs (KDP136) increased the stress fiber formation (*P* = 0.017) compared control cells. There were no differences between *Pg* OMVs (WT) and *Pg* OMVs (KDP136) treatment (*P* = 0.363) (Fig. S1B‐j). *Pg* OMVs (KDP136) also increased Evans blue compared to the control (saline injection) in mice (Fig. S1C). These observations suggested that gingipains did not affect *Pg* OMVs‐induced hyperpermeability, whereas *Pg* OMVs contained enough gingipains to directly digest VEc proteins. Taken together, we conclude that Rho kinases (but not gingipains) play important roles in *Pg* OMVs‐regulated endothelial permeability via stress fiber formation and VEc degradation.

## Discussion

**Fig. 6:**
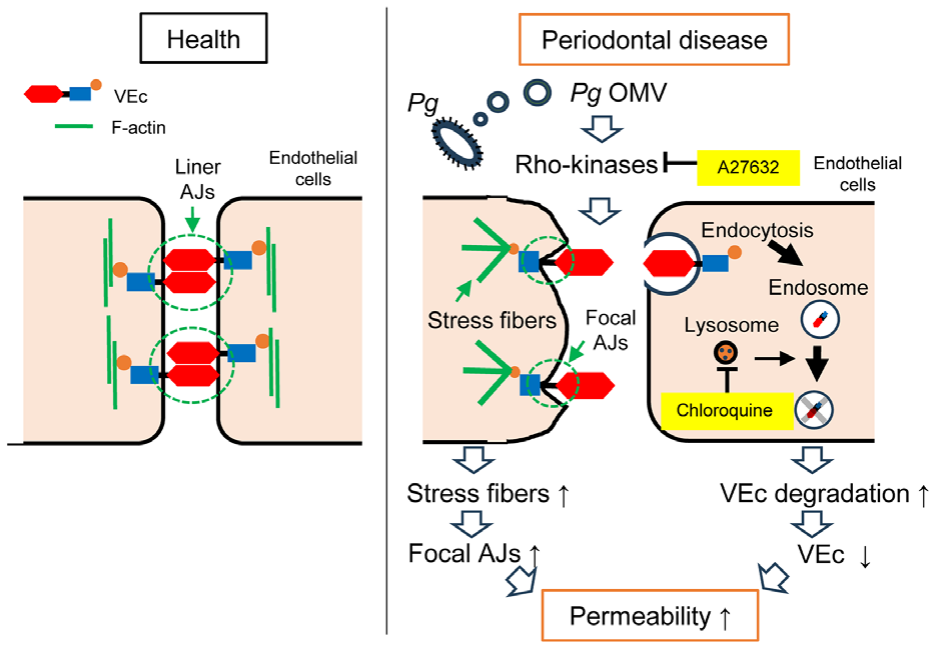
Graphical summary. In healthy individuals, the cell–cell junction is constructed with linear adhesion junctions (AJs) supported by circumferential actin bundles. Therefore, the endothelial barrier was strong and had normal vascular permeability. *Porphyromonas gingivalis* (*Pg*) outer membrane vesicles (OMVs) released during periodontal disease may induce Rho activation. Activated Rho kinases increase stress fiber formation, thereby weakening the endothelial barrier by forming focal AJs. *Pg* OMVs‐activated Rho kinases also promote vascular endothelial‐cadherin (VEc) internalization via endocytosis and subsequent degradation via the endosomal and lysosomal pathways, leading to a decrease in VEc levels.

We showed that *Pg* OMVs increased vascular permeability by primarily modulating two mechanisms in endothelial cells: inducing stress fiber formation and promoting VEc degradation via the lysosomal/endosomal pathway. Rho kinases may be the key factors that regulate both pathways. To the best of our knowledge, this is the first report showing that periodontal bacteria or their products increase hyperpermeability by accelerating cytoskeletal reorganization and inducing lysosomal/endosomal pathway‐mediated protein degradation (Fig. 6).

The gingipains of *Pg* or *Pg* OMVs directly decrease various kinds of endothelial or epithelial adhesion molecules by proteolytic digestion [27, 32, 33]. A recent study indicated that *Pg* gingipains promote vascular permeability by degrading PCAM‐1 while simultaneously increasing bacterial colonization in the liver, kidney, spleen, and lung, owing to reduced leukocyte transendothelial migration capacity [34]. These observations suggest that *Pg* gingipains may be important in modulating vascular permeability in periodontal disease‐associated systemic diseases. The *Pg* OMVs isolated and used in this study contained sufficient active gingipains to degrade VEc proteins immunoprecipitated from HUVEC lysates (Fig. S1A). However, gingipains were not implicated in the decrease of VEc protein levels induced by *Pg* OMVs (Fig. 5D). It is unclear why gingipains were able to cleave immunoprecipitated VE‐cadherin but failed to cleave cellular VE‐cadherin. Therefore, further investigation is required to decipher these discrepancies.

Therefore, we focused on the internalization of VEc by endocytosis and the subsequent degradation of VEc through the lysosomal/endosomal pathway. Chloroquine‐induced lysosomal inhibition markedly enhanced vesicular VEc accumulation in the cytoplasm of *Pg* OMVs‐treated HUVECs. This suggests that *Pg* OMVs increase VEc internalization via endocytosis (Fig. 4A). Most vesicular VEcs were located around the LAMP‐1‐positive structure (indicating lysosomes and late endosomes), and a part of vesicular VEc co‐localized with the LAMP‐1‐positive structure. These observations suggest that internalized VEc is likely targeted for degradation, at least in part, by lysosomes and endosomes. To directly demonstrate that VEc on the cell surface is internalized and metabolized by lysosomes and endosomes, surface levels of VEc in *Pg* OMVs‐treated HUVECs in the presence of chloroquine should be quantified. An inhibitor of endocytosis would have been useful to show that *Pg* OMVs may affect endocytosis.

Inhibition of Rho kinases by A27632 impaired *Pg* OMVs‐induced stress fiber formation and VEc degradation. Therefore, we hypothesized that Rho kinases play a critical role in *Pg* OMV‐induced hyperpermeability. However, it remains unclear which factors in *Pg* OMVs trigger Rho kinase activation. Rho kinases are activated in response to various vasoactive agents such as thrombin, histamine, TNF‐α, and VEGF and induce hyperpermeability by breaking down the endothelial barrier [35]. Many factors can trigger Rho kinase activation because *Pg* OMVs are composed of outer membrane proteins, such as LPS, phospholipids, nucleic acids, and periplasm during their formation [36]. Among these, LPS seems to be a candidate for activating Rho kinases. It has been reported that LPS increases vascular permeability by increasing the expression of Rho kinases. Furthermore, Rho kinase inhibition using A27632 and fasudil alleviated LPS‐induced hyperpermeability in HUVECs and HPMECs [37, 38]. Reports showing that LPS modulates signaling for VEc disruption [39] and stress fiber formation [40, 41] supported our results.

We observed that *Pg* OMVs rapidly increased stress fibers and focal AJs within 30 min after challenge (data not shown), whereas VEc degradation occurred 180 min post*‐Pg* OMVs challenge after a delay in stress fiber and focal AJs formation. It remains unknown whether these two phenomena are linked to each other or whether they are induced separately. Cytoskeletal reorganization, such as stress fiber formation, is thought to influence the loss of cell surface VEc in AJs, because VEc is linked through its cytoplasmic domain to β‐catenin, which in turn binds to α‐catenin, which is anchored to F‐actin bundles [16]. It is necessary to verify whether *Pg* OMVs‐induced stress fibers can modulate signaling to activate lysosomal/endosomal pathways that result in VEc degradation. Furthermore, the reduction in VEc seemed to be marginal in *Pg* OMVs‐treated HUVECs and HPMECs (Fig. 3B). The degree of blood permeability induced by a marginal reduction in VEc should be carefully verified.

Our study did have a few limitations. Our results suggest that Rho kinase is implicated in *Pg* OMVs‐induced stress fiber formation and VEc reduction. Although many studies have reported that gingipains are critical factors in increasing vascular permeability, we observed some discrepancies in this regard, therefore further studies using gingipain inhibitors are required to verify the role of these proteases in VE‐cadherin cleavage and permeability. Furthermore, vascular permeability was assessed using the Miles assay and transwell system in this study; future studies should perform quantitative evaluation, for example, quantifying Miles assay and measuring trans‐epithelial electrical resistance.

Many oral bacteria, including periodontal pathogens, produce OMVs with various biological effects [37]. To verify the specificity of *Pg* OMVs in inducing vascular permeability, the functions of other bacterial OMVs in stress fiber formation and VEc degradation should be assessed in the future.

Virulence factors wrapped in OMVs have various advantages, including protection from proteolytic degradation and enhanced distance delivery [42]. We observed that *Pg* OMVs can deliver gingipains to distant organs, such as the liver and brain [11, 12]. The ability of *Pg* OMVs to disrupt the endothelial barrier, as shown in this study, may facilitate *Pg* OMV translocation to distant organs by promoting its infiltration and leakage through blood vessels. This new perspective will help us better understand the pathogenesis of periodontal disease‐associated systemic diseases.

## Materials and methods

### Materials and antibodies

All reagents used in this study were commercially available, including 70 kDa fluorescein isothiocyanate (FITC)‐labeled dextran (46945; Sigma, St. Louis, MO, USA), chloroquine (AG‐CR1‐3721; AdipoGen Life Sciences, San Diego, CA, USA), A27632 (HY‐10071; MedChemExpress, Monmouth Junction, NJ, USA), and Protein A/G PLUS‐agarose (sc‐2003; Santa Cruz Biotechnology Inc., Santa Cruz, CA, USA).

The following primary antibodies were used: VEc (1:1000, #2500; Cell Signaling Technology, Beverly, MA, USA), phospho‐cofilin (Ser3) (77G2) (1:1000, #3313; Cell Signaling Technology), cofilin (D3F9) (1:1000, #5175; Cell Signaling Technology), GAPDH antibody (14C10) (1:1000, #2118), and LAMP‐1 (1:100, Genetex, GT25212).

### Bacterial cultures

*Pg* ATCC33277 was cultured in Brain Heart infusion medium (BD Bioscience, Franklin Lakes, NJ, USA) containing 0.5% yeast extract (BD Bioscience), 10 μg·mL^−1^ hemin (Wako Chemicals, Osaka, Japan), 1 μg·mL^−1^ 2‐methyl‐1, 4‐naphthoquinone (Tokyokasei, Tokyo, Japan). Rgp and Kgp gingipain‐deficient *Pg* ATCC 33277 (KDP136) was kindly provided by Dr. Koji Nakayama (Nagasaki University) and cultured in the medium with 25 μg·mL^−1^ chloramphenicol, 10 μg·mL^−1^ erythromycin, and 1 μg·mL^−1^ tetracycline. All strains were cultured in an anaerobic jar at 37 °C.

### Isolation of OMVs

OMVs were isolated as previously described [11, 43]. Briefly, bacterial culture medium was centrifuged at 2800 ***g*** for 15 min at 4 °C. The supernatant was collected and filtered with a 0.2‐μm syringe filter and concentrated using an Ultra‐15 Centrifugal Filter Device with a nominal molecular weight limit of 100 000 (Merck Millipore, Burlington, MA, USA). OMVs were isolated from the concentrate using the Total Exosome Isolation Reagent (Thermo Fisher Scientific, Waltham, MA, USA) according to the manufacturer's protocol. The protein concentration of OMVs used for the experiments was measured using Bradford Protein Assay Reagent (Bio‐Rad Laboratories Inc., Hercules, CA, USA).

In a previous study, we confirmed that OMVs were stably present in samples using multiple methods, such as scanning electron microscopy and transmission electron microscopy, and by measuring the diameter of *Pg* OMVs [11]. The isolated *Pg* OMVs were stored at 4 °C and used within a week. The concentration of the *Pg* OMVs was measured and adjusted immediately before use.

### Cell culture

Human primary umbilical vein endothelial cells (HUVECs) (C2519A; Lonza Walkersville, MD, USA) were obtained from Lonza Walkersville and cultured using an EGMTM‐2 Bullet Kit (CC‐3126; Lonza Walkersville). Human primary pulmonary microvascular endothelial cells (HPMECs) (C‐12281; PromoCell GmbH, Heidelberg, Germany) were purchased from PromoCell and maintained in MV medium prepared using an Endothelial Cell Growth Medium MV Kit (C‐22121; PromoCell). HUVECs and HPMECs used in the experiments were authenticated based on high expression of VEc protein and cell morphology and were verified to be mycoplasma‐free. All cells were cultured at 37 °C under a humidified atmosphere of 5% CO_2_ and used for experiments after reaching 70–80% confluence.

### Vascular permeability

BALB/cAJc1 mice (female, 40 weeks old), purchased from CLEA Japan (Tokyo, Japan), were used for the Miles assay (29). Mice were kept in plastic gages and maintained at ambient temperature 22–24 °C under a 12 h light/dark cycle and fed a standard solid diet with water *ad libitum*. *Pg* OMVs containing 15 μg protein or saline were injected into dorsal skin of mice, followed by injection of 100 μL of 0.5% Evans blue into the tail veins. *Pg* OMVs/saline was administered as a single injection. After 10 min, the gross Evans blue dye level on the skin surface was visually observed. The experiments were performed three times using four mice in each group, and a typical image of visual discoloration is shown. All animal experiments were approved by the Ethics Committee of Animal Care and Experimentation of Tokushima University (approval number: T29‐31).

The permeability of the endothelial cell monolayer was measured using a Transwell assay system (Merck Millipore, Watford, UK). HUVECs or HPMECs were seeded onto culture inserts and cultured until they reached confluence. Subsequently, the cells were treated with or without *Pg* OMVs at 500 ng·mL^−1^ for 1 h with or without the inhibitor. FITC‐labeled dextran (1 mg·mL^−1^) was added to the apical compartment of the insert and incubated for 10 min. Leakage of FITC‐labeled dextran from the apical compartment of the insert to the bottom well was quantified by measuring the fluorescence intensity (excitation, 494 nm; emission, 521 nm) using a plate reader (Infinite 200 PRO, TECAN). All experiments were performed using 4–5 wells for each group in three independent experiments.

### Immunofluorescence analysis

HUVECs or HPMECs were fixed in 4% formalin for 30 min and permeabilized with 0.1% Triton X‐100 in phosphate‐buffered saline (PBS) for 2 min on ice. The cells were blocked with 4% bovine serum albumin (BSA) in PBS for 45 min, and the cells were incubated with anti‐VEc antibody (D87F2, #2500; Cell Signaling Technology) or normal rabbit IgG (#2729; Cell Signaling Technology) overnight at 4 °C, followed by incubation with Alexa Fluor 566‐conjugated anti‐rabbit IgG (Thermo Fisher Scientific) for 60 min. The cells were then treated with 40 μL·mL^−1^ FITC‐phalloidin (ActinGreen™ 488; Thermo Fisher Scientific) for 1 h, followed by treatment with 500 μg·mL^−1^ Hoechst 33342 for 30 min for nuclear staining. The samples were mounted and observed under a BZ‐X800 fluorescence microscope (Keyence, Osaka, Japan) or a Nikon A1R confocal microscope (Nikon, Tokyo, Japan).

FITC‐phalloidin‐labeled F‐actin from the images of four samples per experimental condition was quantified using a BZ‐X800 Analyzer (Keyence) with a Hybrid Cell Count system. The fluorescence intensity was analyzed using Student's *t*‐test and is quantitatively represented in a graph.

### Lysosomal inhibition and vesicular VEc detection

HUVECs were cultured and pretreated with 100 μm chloroquine for 30 min, followed by challenge with or without 500 ng·mL^−1^ of *Pg* OMVs for 3 h. The cells were fixed with 4% formalin and permeabilized with 0.1% Triton X‐100 in PBS for 2 min on ice. Subsequently, the cells were blocked with 4% BSA in PBS for 45 min and incubated with anti‐VEc antibody overnight at 4 °C. The cells were then incubated with Alexa Fluor 566‐conjugated anti‐rabbit IgG (Thermo Fisher Scientific) for 60 min and mounted and observed under a BZ‐X800 fluorescence microscope (Keyence).

### Quantification and co‐localization of VEc and LAMP‐1

HUVECs stained with VEc antibody and Alexa Fluor 566‐conjugated anti‐rabbit IgG as described above were incubated with anti‐LAMP‐1 antibody overnight at 4 °C consecutively. After washing, the cells were treated with Alexa Fluor 488‐conjugated anti‐mouse IgG for 60 min, and the nuclei were stained with Hoechst 33342 for 20 min. Subsequently, the cells were mounted on glass slides and observed under a BZ‐X800 microscope as described above.

To detect co‐localization of VEc and LAMP‐1 in HUVECs, the fluorescence of Alexa 566‐labeled VEc (red) and Alexa 488‐labeled LAMP‐1 (green) was merged in the images. In the fields where vesicular VEc was observed around the nucleus, the merged yellow and green fluorescent intensities were quantified using a BZ‐X800 Analyzer (Keyence) with a Hybrid Cell Count system. The co‐localization of VEc and LAMP‐1 was analyzed by calculating the ratio of merged (yellow) and LAMP‐1 (green) fluorescent intensities and are shown as co‐localization coefficients.

### Western blot analysis

Cells were scraped into lysis buffer (1 mm dithiothreitol (DTT), 0.2 mm phenylmethylsulphonyl fluoride (PMSF), 1 μg·mL^−1^ leupeptin, 4 μg·mL^−1^ aprotinin, and 50 mm NaF) and centrifuged at 12 000 ***g*** for 30 min. The supernatants were separated using sodium dodecyl sulfate‐polyacrylamide gel electrophoresis (SDS/PAGE) and transferred to polyvinylidene difluoride membranes (Immobilon‐P, Millipore, Temecula, CA, USA). The membranes were incubated with primary antibodies for 12 h at 4 °C, washed with TBS‐T for 30 min, and subsequently incubated with anti‐rabbit IgG and HRP‐linked antibodies (1:10 000, #7074; Cell Signaling Technology) for 45 min. The signals were detected using western blot Chemiluminescence HRP substrate (Takara Bio Inc., Otsu, Japan). The band densities of VEc and GAPDH were quantified using ImageJ software (U.S. National Institutes of Health, Bethesda, MD, USA), and the levels of VEc proteins were normalized to GAPDH.

### RNA isolation and real‐time PCR

Cells were homogenized in ISOGEN (Nippon Gene, Tokyo, Japan), and total RNA was isolated according to the manufacturer's protocol. cDNA was synthesized using ReverTra Ace qPCR RT Master Mix (TOYOBO, Kyoto, Japan). Real‐time PCR was performed with a 7300 Real‐Time PCR system (Applied Biosystems, Carlsbad, CA, USA) using THUNDERBIRD SYBR qPCR Mix (TOYOBO). The following primer sequences were used: human VE‐cadherin (NM_001130861.1): forward, 5′‐CTGCCCTTAACAGACGGAATGAA‐3′, reverse, 5′‐ACCCGCTCTGCCTATGGAAAC‐3′; human GAPDH (NM_002046.7): forward, 5′‐GCACCGTCAAGGCTGAGAAC‐3′, reverse, 5′‐TGGTGAAGACGCCAGTGGA‐3′.

### Immunoprecipitation of VE‐cadherin

Human umbilical vein endothelial cells were homogenized in lysis buffer (1 mm DTT, 0.2 mm PMSF, 1 μg·mL^−1^ leupeptin, 4 μg·mL^−1^ aprotinin, and 50 mm NaF) and centrifuged at 12 000 ***g*** for 20 min at 4 °C. The supernatant was rotated for 4 h at 4 °C with rabbit anti‐VEc antibody (1:100), and further incubated with Protein A/G PLUS‐Agarose for 12 h at 4 °C. The VEc‐coupled beads were washed at least five times with lysis buffer.

### VE‐cadherin degradation by *Pg* OMVs

The obtained VEc‐coupled beads were suspended in reaction buffer (0.1 m Tris/HCl pH 7.6, 50 mm NaCl, and 5 mm CaCl_2_). An aliquot was incubated with 3 μg protein of *Pg* OMVs or 5 μL of *Pg* cultured medium for 10 min at 37 °C. The reaction was stopped by the addition of 5× Laemmli sample buffer (1 m Tris/HCl pH 6.8, 0.5 m Dithiothreitol, 10% SDS, 50% glycerol, 0.02% bromophenol blue) and boiled for 5 min. Samples were analyzed for VEc degradation using western blotting.

### Statistical analysis

Statistical analyses were performed using the statcel2 software. The normal distribution of the data was first examined using the chi‐square test. Variables with a normal distribution were analyzed using Student's *t*‐test. All data are expressed as the mean ± standard deviation (SD).

## Conflict of interest

The authors declare no conflict of interest.

## Author contributions

KY and KO: Conceptualization. KY: Project administration. KO: Supervision. KY: Funding acquisition. MM, AT, MS, KY, and AI: Investigation. KY and YH: Methodology. MM, KY, and YH: Writing.

### Peer review

The peer review history for this article is available at https://www.webofscience.com/api/gateway/wos/peer‐review/10.1111/febs.17349.

## Supporting information


**Fig. S1.** *Pg* OMVs induced increase in permeability is unaffected by gingipains.

**Material S1.** Quantification of stress fibers in HUVECs (Figure 2E).
**Material S2.** Quantification of stress fibers in HPMECs (Fig. 2E).
**Material S3.** Co‐localization of VEcand LAMP‐1 (Fig. 4B‐i).
**Material S4.** Involvement of Rho A kinase (Fig. 5C).
**Material S5.** Effects of gingipains (Fig. S1B).

## Data Availability

The data that support the findings of this study are available from the corresponding author upon reasonable request.

## References

[1] Kanagasingam S , Chukkapalli SS , Welbury R & Singhrao SK (2020) *Porphyromonas gingivalis* is a strong risk factor for Alzheimer's disease. J Alzheimers Dis Rep 4, 501–511. 33532698 10.3233/ADR-200250 PMC7835991

[2] Watanabe K , Katagiri S , Takahashi H , Sasaki N , Maekawa S , Komazaki R , Hatasa M , Kitajima Y , Maruyama Y , Shiba T *et al*. (2021) *Porphyromonas gingivalis* impairs glucose uptake in skeletal muscle associated with altering gut microbiota. FASEB J 35, e21171. 33197074 10.1096/fj.202001158R

[3] Bielaszewska M , Daniel O , Nyč O & Mellmann A (2021) In vivo secretion of β‐lactamase‐carrying outer membrane vesicles as a mechanism of β‐lactam therapy failure. Membranes 11, 806. 34832035 10.3390/membranes11110806 PMC8625792

[4] Ishikawa M , Yoshida K , Okamura H , Ochiai K , Takamura H , Fujiwara N & Ozaki K (2013) Oral *Porphyromonas gingivalis* translocates to the liver and regulates hepatic glycogen synthesis through the Akt/GSK‐3beta signaling pathway. Biochim Biophys Acta 1832, 2035–2043. 23899607 10.1016/j.bbadis.2013.07.012

[5] Veith PD , Chen YY , Gorasia DG , Chen D , Glew MD , O'Brien‐Simpson NM , Cecil JD , Holden JA & Reynolds EC (2014) *Porphyromonas gingivalis* outer membrane vesicles exclusively contain outer membrane and periplasmic proteins and carry a cargo enriched with virulence factors. J Proteome Res 13, 2420–2432. 24620993 10.1021/pr401227e

[6] Inaba H , Kawai S , Kato T , Nakagawa I & Amano A (2006) Association between epithelial cell death and invasion by microspheres conjugated to *Porphyromonas gingivalis* vesicles with different types of fimbriae. Infect Immun 74, 739. 10.1128/IAI.74.1.734-739.2006 PMC1346634 16369031

[7] Mantri CK , Chen CH , Dong X , Goodwin JS , Pratap S , Paromov V & Xie H (2015) Fimbriae‐mediated outer membrane vesicle production and invasion of *Porphyromonas gingivalis* . Microbiology 4, 53–65. 10.1002/mbo3.221 PMC4335976 25524808

[8] Bomberger JM , Maceachran DP , Coutermarsh BA , Ye S , O'Toole GA & Stanton BA (2009) Long‐distance delivery of bacterial virulence factors by *Pseudomonas aeruginosa* outer membrane vesicles. PLoS Pathog 5, e1000382. 19360133 10.1371/journal.ppat.1000382 PMC2661024

[9] Zhang Z , Liu D , Liu S , Zhang S & Pan Y (2020) The role of *Porphyromonas gingivalis* outer membrane vesicles in periodontal disease and related systemic diseases. Front Cell Infect Microbiol 10, 585917. 33585266 10.3389/fcimb.2020.585917 PMC7877337

[10] Gong T , Chen Q , Mao H , Zhang Y , Ren H , Xu M , Chen H & Yang D (2022) Outer membrane vesicles of *Porphyromonas gingivalis* trigger NLRP3 inflammasome and induce neuroinflammation, tau phosphorylation, and memory dysfunction in mice. Front Cell Infect Microbiol 12, 925435. 36017373 10.3389/fcimb.2022.925435 PMC9397999

[11] Seyama M , Yoshida K , Yoshida K , Fujiwara N , Ono K , Eguchi T , Kawai H , Guo J , Weng Y , Haoze Y *et al*. (2020) Outer membrane vesicles of *Porphyromonas gingivalis* attenuate insulin sensitivity by delivering gingipains to the liver. Biochim Biophys Acta Mol Basis Dis 1866, 165731. 32088316 10.1016/j.bbadis.2020.165731

[12] Yoshida K , Yoshida K , Seyama M , Hiroshima Y , Mekata M , Fujiwara N , Kudo Y & Ozaki K (2022) *Porphyromonas gingivalis* outer membrane vesicles in cerebral ventricles activate microglia in mice. Oral Dis 29, 3688–3697. 36266256 10.1111/odi.14413

[13] Komarova Y & Malik AB (2010) Regulation of endothelial permeability via paracellular and transcellular transport pathways. Annu Rev Physiol 72, 463–493. 20148685 10.1146/annurev-physiol-021909-135833

[14] Komarova YA , Kruse K , Mehta D & Malik AB (2017) Protein interactions at endothelial junctions and signaling mechanisms regulating endothelial permeability. Circ Res 120, 179–206. 28057793 10.1161/CIRCRESAHA.116.306534 PMC5225667

[15] Taddei A , Giampietro C , Conti A , Orsenigo F , Breviario F , Pirazzoli V , Potente M , Daly C , Dimmeler S & Dejana E (2008) Endothelial adherens junctions control tight junctions by VE‐cadherin‐mediated upregulation of claudin‐5. Nat Cell Biol 10, 923–934. 18604199 10.1038/ncb1752

[16] Dejana E , Orsenigo F & Lampugnani MG (2008) The role of adherens junctions and VE‐cadherin in the control of vascular permeability. J Cell Sci 121, 2115–2122. 18565824 10.1242/jcs.017897

[17] Shen D , Ye X , Li J , Hao X , Jin L , Jin Y , Tong L & Gao F (2022) Metformin preserves VE‐cadherin in choroid plexus and attenuates hydrocephalus via VEGF/VEGFR2/p‐Src in an intraventricular hemorrhage rat model. Int J Mol Sci 23, 8552. 35955686 10.3390/ijms23158552 PMC9369137

[18] Xiao K , Allison DF , Kottke MD , Summers S , Sorescu GP , Faundez V & Kowalczyk AP (2003) Mechanisms of VE‐cadherin processing and degradation in microvascular endothelial cells. J Biol Chem 278, 19199–19208. 12626512 10.1074/jbc.M211746200

[19] Xiao K , Garner J , Buckley KM , Vincent PA , Chiasson CM , Dejana E , Faundez V & Kowalczyk AP (2005) p120‐catenin regulates clathrin‐dependent endocytosis of VE‐cadherin. Mol Biol Cell 16, 5141–5151. 16120645 10.1091/mbc.E05-05-0440 PMC1266414

[20] Rho SS , Ando K & Fukuhara S (2017) Dynamic regulation of vascular permeability by vascular endothelial cadherin‐mediated endothelial cell‐cell junctions. J Nippon Med Sch 84, 148–159. 28978894 10.1272/jnms.84.148

[21] Yamamoto K , Takagi Y , Ando K & Fukuhara S (2021) Rap1 small GTPase regulates vascular endothelial‐cadherin‐mediated endothelial cell‐cell junctions and vascular permeability. Biol Pharm Bull 44, 1371–1379. 34602545 10.1248/bpb.b21-00504

[22] Dorard C , Cseh B , Ehrenreiter K , Wimmer R , Varga A , Hirschmugl T , Maier B , Kramer K , Fürlinger S , Doma E *et al*. (2019) RAF dimers control vascular permeability and cytoskeletal rearrangements at endothelial cell‐cell junctions. FEBS J 286, 2277–2294. 30828992 10.1111/febs.14802 PMC6617973

[23] van Nieuw Amerongen GP , Beckers CM , Achekar ID , Zeeman S , Musters RJ & van Hinsbergh VW (2007) Involvement of Rho kinase in endothelial barrier maintenance. Arterioscler Thromb Vasc Biol 27, 2332–2339. 17761936 10.1161/ATVBAHA.107.152322

[24] Oldenburg J & de Rooij J (2014) Mechanical control of the endothelial barrier. Cell Tissue Res 355, 545–555. 24519624 10.1007/s00441-013-1792-6

[25] Farrugia C , Stafford GP & Murdoch C (2020) *Porphyromonas gingivalis* outer membrane vesicles increase vascular permeability. J Dent Res 99, 1494–1501. 32726180 10.1177/0022034520943187 PMC7684789

[26] Huang S , Cao G , Dai D , Xu Q , Ruiz S , Shindo S , Nakamura S , Kawai T , Lin J & Han X (2023) *Porphyromonas gingivalis* outer membrane vesicles exacerbate retinal microvascular endothelial cell dysfunction in diabetic retinopathy. Front Microbiol 14, 1167160. 37250057 10.3389/fmicb.2023.1167160 PMC10213754

[27] Nonaka S , Kadowaki T & Nakanishi H (2022) Secreted gingipains from *Porphyromonas gingivalis* increase permeability in human cerebral microvascular endothelial cells through intracellular degradation of tight junction proteins. Neurochem Int 154, 105282. 35032577 10.1016/j.neuint.2022.105282

[28] Eguchi T , Sogawa C , Okusha Y , Uchibe K , Iinuma R , Ono K , Nakano K , Murakami J , Itoh M , Arai K *et al*. (2018) Organoids with cancer stem cell‐like properties secrete exosomes and HSP90 in a 3D nanoenvironment. PLoS One 13, e0191109. 29415026 10.1371/journal.pone.0191109 PMC5802492

[29] Park‐Windhol C & D'Amore PA (2016) Disorders of vascular permeability. Annu Rev Pathol 11, 251–281. 26907525 10.1146/annurev-pathol-012615-044506 PMC8462517

[30] Mauthe M , Orhon I , Rocchi C , Zhou X , Luhr M , Hijlkema KJ , Coppes RP , Engedal N , Mari M & Reggiori F (2018) Chloroquine inhibits autophagic flux by decreasing autophagosome‐lysosome fusion. Autophagy 14, 1435–1455. 29940786 10.1080/15548627.2018.1474314 PMC6103682

[31] Marsh M , Schmid S , Kern H , Harms E , Male P , Mellman I & Helenius A (1987) Rapid analytical and preparative isolation of functional endosomes by free flow electrophoresis. J Cell Biol 104, 875–886. 3031085 10.1083/jcb.104.4.875 PMC2114435

[32] Maekawa M , Ishizaki T , Boku S , Watanabe N , Fujita A , Iwamatsu A , Obinata T , Ohashi K , Mizuno K & Narumiya S (1999) Signaling from Rho to the actin cytoskeleton through protein kinases ROCK and LIM‐kinase. Science 285, 895–898. 10436159 10.1126/science.285.5429.895

[33] Farrugia C , Stafford GP , Potempa J , Wilkinson RN , Chen Y , Murdoch C & Widziolek M (2020) Mechanisms of vascular damage by systemic dissemination of the oral pathogen *Porphyromonas gingivalis* . FEBS J 288, 1479–1495. 32681704 10.1111/febs.15486 PMC9994420

[34] Katz J , Yang QB , Zhang P , Potempa J , Travis J , Michalek SM & Balkovetz DF (2002) Hydrolysis of epithelial junctional proteins by *Porphyromonas gingivalis* gingipains. Infect Immun 70, 2512–2518. 11953390 10.1128/IAI.70.5.2512-2518.2002 PMC127922

[35] Zou Z , Fang J , Ma W , Guo J , Shan Z , Ma D , Hu Q , Wen L & Wang Z (2023) *Porphyromonas gingivalis* gingipains destroy the vascular barrier and reduce CD99 and CD99L2 expression to regulate Transendothelial migration. Microbiol Spectr 11, e0476922. 37199607 10.1128/spectrum.04769-22 PMC10269447

[36] Wojciak‐Stothard B & Ridley AJ (2002) Rho GTPases and the regulation of endothelial permeability. Vascul Pharmacol 39, 187–199. 12747959 10.1016/s1537-1891(03)00008-9

[37] Cecil JD , Sirisaengtaksin N , O'Brien‐Simpson NM & Krachler AM (2019) Outer membrane vesicle‐host cell interactions. Microbiol Spectr 7, 10–1128. 10.1128/microbiolspec.psib-0001-2018 PMC6352913 30681067

[38] Wang J , Xu J , Zhao X , Xie W , Wang H & Kong H (2018) Fasudil inhibits neutrophil‐endothelial cell interactions by regulating the expressions of GRP78 and BMPR2. Exp Cell Res 365, 97–105. 29481792 10.1016/j.yexcr.2018.02.026

[39] Xie K , Wang W , Chen H , Han H , Liu D , Wang G & Yu Y (2015) Hydrogen‐rich medium attenuated lipopolysaccharide‐induced monocyte‐endothelial cell adhesion and vascular endothelial permeability via Rho‐associated coiled‐coil protein kinase. Shock 44, 58–64. 25895142 10.1097/SHK.0000000000000365

[40] Chan YH , Harith HH , Israf DA & Tham CL (2019) Differential regulation of LPS‐mediated VE‐cadherin disruption in human endothelial cells and the underlying signaling pathways: a mini review. Front Cell Dev Biol 7, 280. 31970155 10.3389/fcell.2019.00280 PMC6955238

[41] Siddiqui MR , Akhtar S , Shahid M , Tauseef M , McDonough K & Shanley TP (2019) miR‐144‐mediated inhibition of ROCK1 protects against LPS‐induced lung endothelial hyperpermeability. Am J Respir Cell Mol Biol 61, 257–265. 30811958 10.1165/rcmb.2018-0235OC

[42] Xiaolu D , Jing P , Fang H , Lifen Y , Liwen W , Ciliu Z & Fei Y (2011) Role of p115RhoGEF in lipopolysaccharide‐induced mouse brain microvascular endothelial barrier dysfunction. Brain Res 1387, 1–7. 21354111 10.1016/j.brainres.2011.02.059

[43] Bonnington KE & Kuehn MJ (2014) Protein selection and export via outer membrane vesicles. Biochim Biophys Acta 1843, 1612–1619. 24370777 10.1016/j.bbamcr.2013.12.011 PMC4317292

