# Supplementary material for: Porphyromonas gingivalis outer membrane vesicles increase vascular permeability by inducing stress fiber formation and degrading vascular endothelial‐cadherin in endothelial cells
Source: FEBS J. 2024 Dec 17;292(7):1696–709. doi: 10.1111/febs.17349 (PMC11970716; doi:10.1111/febs.17349)
Supplement: Supplementary file 1 — Fig. S1. Pg OMVs induced increase in permeability is unaffected by gingipains. [file FEBS-292-1696-s002.docx]

***Porphyromonas gingivalis* outer membrane vesicles increase vascular permeability by inducing stress fiber formation and vascular endothelial-cadherin degradation in endothelial cells**

Mana Mekata^1^, Kaya Yoshida^1^*, Ayu Takai^1^, Yuka Hiroshima^2^, Ayu Ikuta^1^, Mariko Seyama^1^, Kayo Yoshida^1^, Kazumi Ozaki^1^.

**Corresponding author:** Kaya Yoshida, DDS, Ph.D.

3-18-15, Kuramoto, Tokushima 770-8504, Japan.

TEL: 81-88-633-7898, FAX: 81-88-633-7898; E-mail: kaya@tokushima-u.ac.jp

ORCID ID: <https://orcid.org/0000-0002-3202-0634>

**Supporting Information**


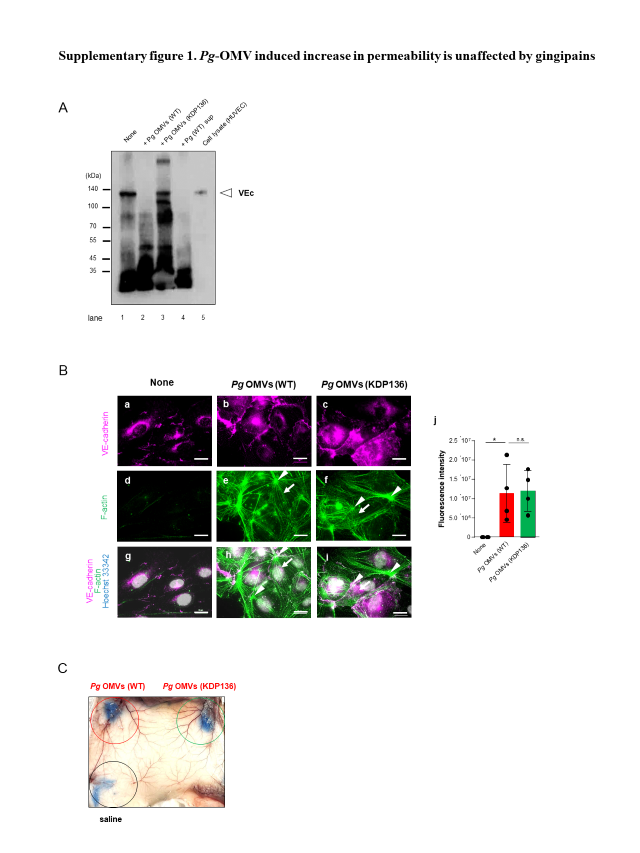


Supplementary figure 1. ***Pg*-OMV induced increase in permeability is unaffected by gingipains**

(A) Vascular endothelial-cadherin (VEc) immunoprecipitated from human umbilical vein endothelial cells (HUVECs) was incubated with wild type- *Porphyromonas gingivalis (Pg*) outer membrane vesicles (OMVs) (WT) or gingipain-deficient *Pg* OMVs (KDP136) or cultured media of *Pg* (WT) (*Pg* sup). Subsequently, VEc degradation was assessed via western blotting. (B) HUVECs were treated with *Pg* OMVs (WT) (b, e, h) or *Pg* OMVs (KDP136) (c, f, i) for 60 min. Cells were fixed and stained as described in Figure 2. Arrows indicate the radical stress fibers. The arrowhead shows adhesion junctions (AJs) connected to stress fibers (e, f, h, i). Scale bars represent 20 μm. The fluorescence intensity of F-actin (d, e, f) was quantified (i). The Student’s *t*-test was used for statistical analysis (n=4 in each group). Error bars represent standard deviation. * *p* < 0.05 compared with none. n. s. indicate not significant compared with *Pg* OMVs (WT) (C) Representative dorsal skin images of mice in Miles assay. Fifteen micrograms of *Pg* OMVs (WT) (red circle) or saline (black circle) were injected into the right side of the skin, and *Pg* OMVs (KDP136) were injected into the left side (green circle).

AJs, adhesion junctions; HUVECs, human umbilical vein endothelial cells; OMV, outer membrane vesicle; *Pg*, *Porphyromonas gingivalis*; VEc, vascular endothelial-cadherin; WT, wild-type
