# Supplementary material for: Porphyromonas gingivalis outer membrane vesicles increase vascular permeability by inducing stress fiber formation and degrading vascular endothelial‐cadherin in endothelial cells
Source: FEBS J. 2024 Dec 17;292(7):1696–709. doi: 10.1111/febs.17349 (PMC11970716; doi:10.1111/febs.17349)
Supplement: Supplementary file 2 — Material S1. Quantification of stress fibers in HUVECs (Figure 2E). Material S2. Quantification of stress fibers in HPMECs (Fig. 2E). Material S3. Co‐localization of VEcand LAMP‐1 (Fig. 4B‐i). Material S4. Involvement of Rho A kinase (Fig. 5C). Material S5. Effects of gingipains (Fig. S1B). [file FEBS-292-1696-s001.pdf]

Explanatory material 1: Quantification of stress fibers in HUVECs (**Figure 2E**)

1st

None

n=3, samples in each group

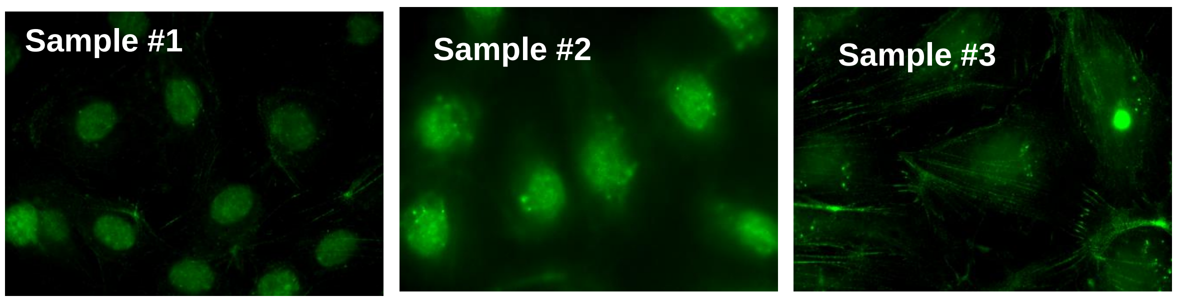

***Pg* OMVs (60 min)**

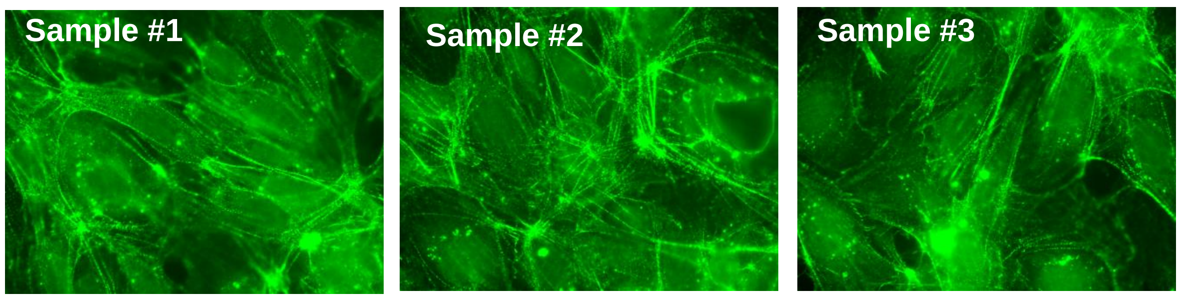

Fluorescence intensity

Group	Sample	Green (F-actin)	mean
None	#1	783509	**1070376**
	#2	450980	
	#3	1976640	
Pg OMVs	#1	6548939	**6846790**
	#2	7747389	
	#3	6244043	

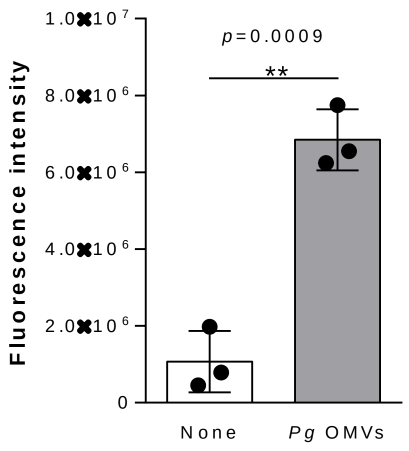

2nd

None

n=4, samples in each group

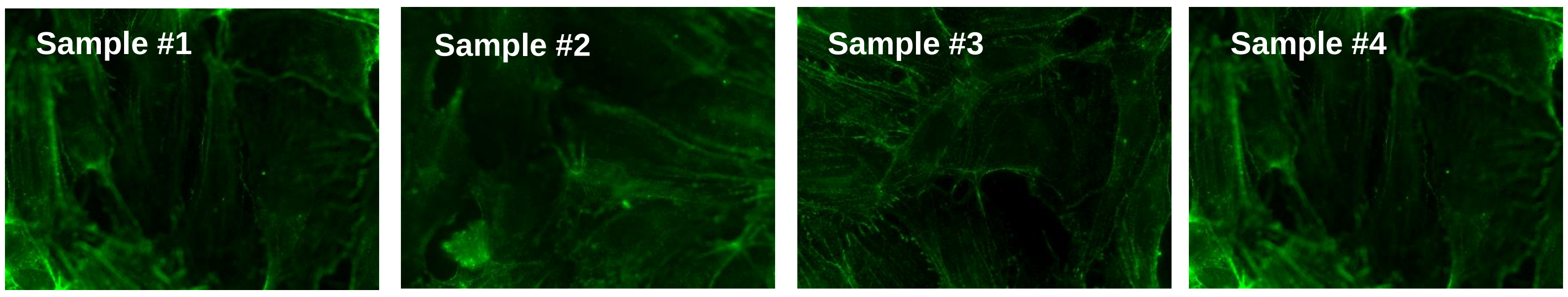

***Pg* OMVs (60 min)**

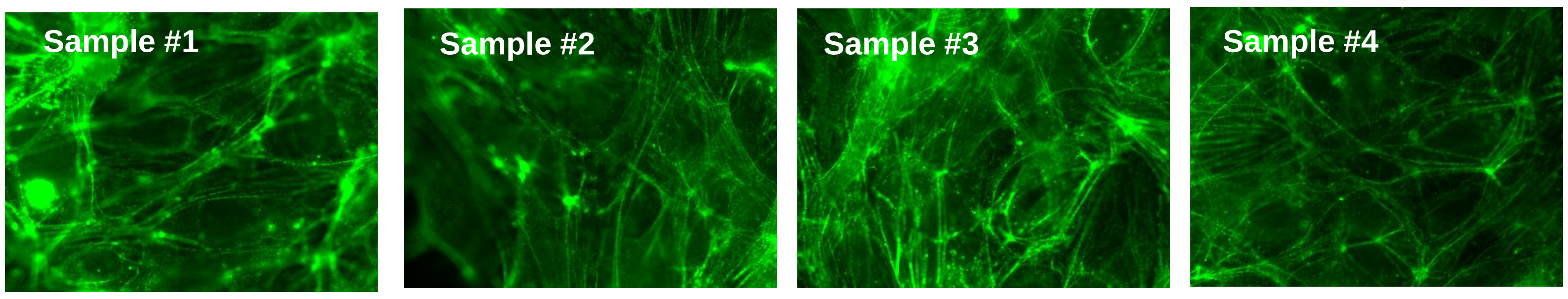

Fluorescence intensity

Group	Sample	Green (F-actin)	mean
None	#1	506270	**261300**
	#2	136974	
	#3	42336	
	#4	359619	
Pg OMVs	#1	9403785	**5400245**
	#2	2963223	
	#3	7835706	
	#4	1398267	

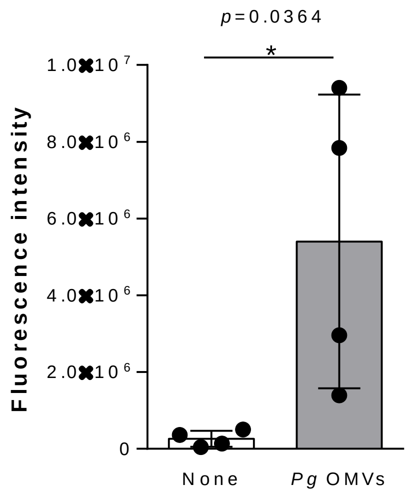

3rd

None

n=4, samples in each group

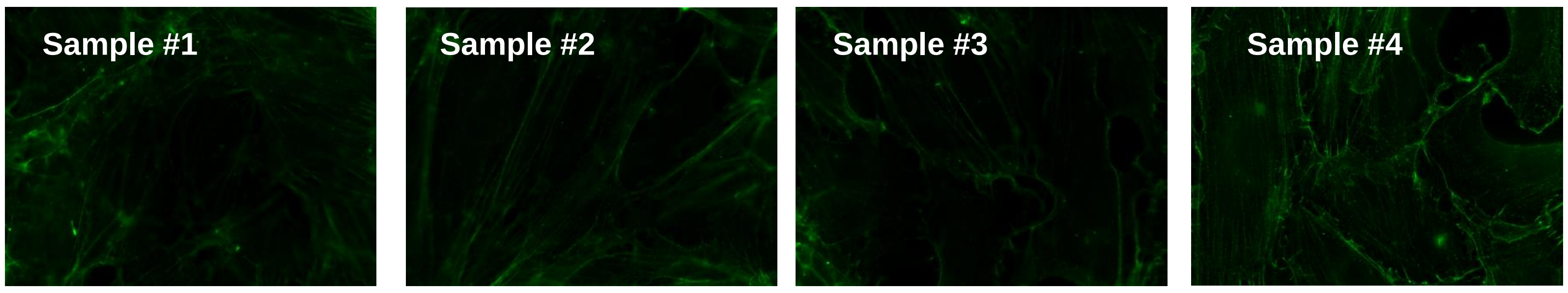

***Pg* OMVs (60 min)**

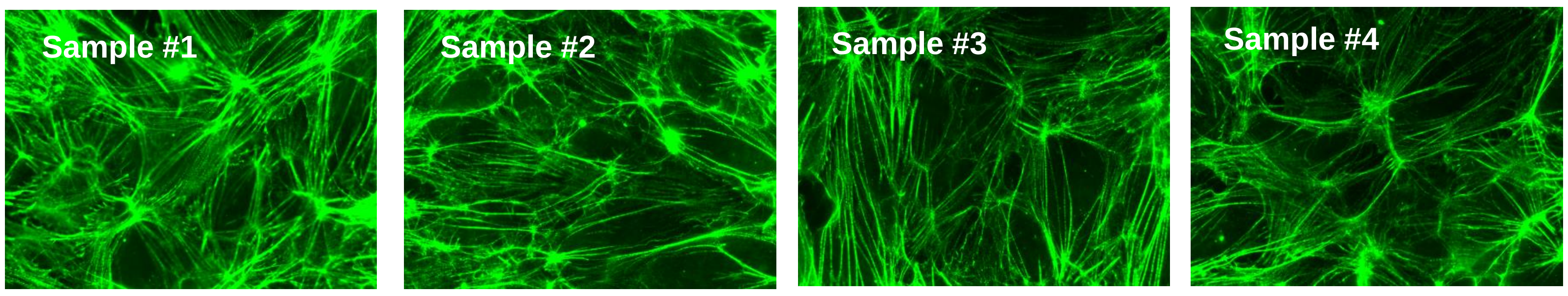

Fluorescence intensity

Group	Sample	Green (F-actin)	mean
None	#1	60385	**55217**
	#2	25678	
	#3	31794	
	#4	103010	
Pg OMVs	#1	18614283	**13111180**
	#2	13936423	
	#3	10707923	
	#4	9186091	

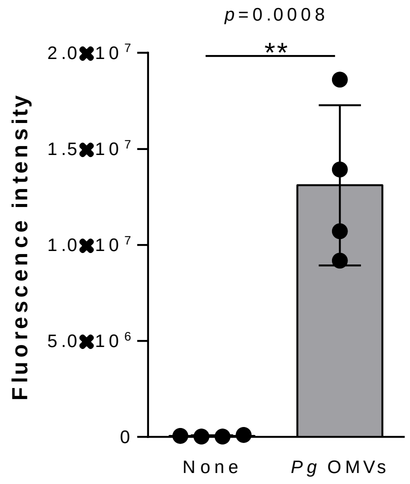

Explanatory material 2: Quantification of stress fibers in HPMECs (**Figure 2E**)

1st

None

n=4 samples (from different cover glasses) in each group

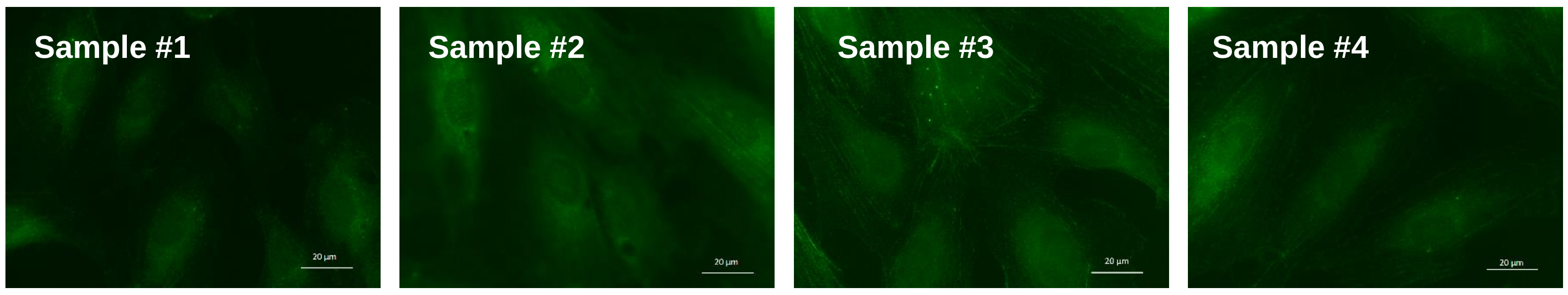

***Pg* OMVs (60 min)**

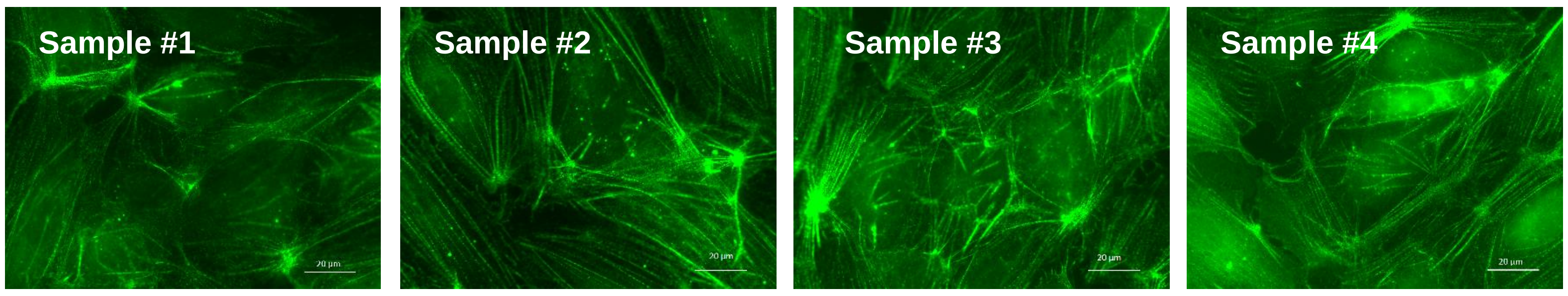

Fluorescence intensity

Group	Sample	Green (F-actin)	Mean ± s.d.
None	#1	12341	**194841**
	#2	428002	
	#3	250887	
	#4	419701	
Pg OMVs	#1	10935671	**14991990**
	#2	17656329	
	#3	35283606	
	#4	43141127	

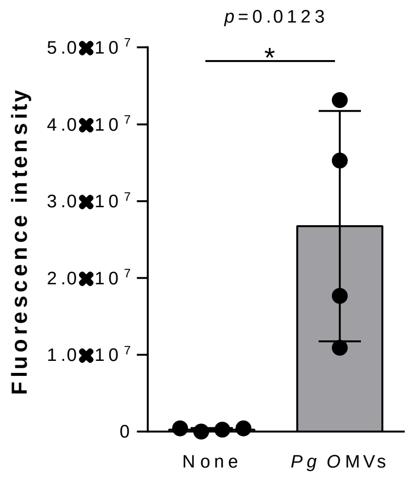

2nd

None

n=4 samples (from different cover glasses) in each group

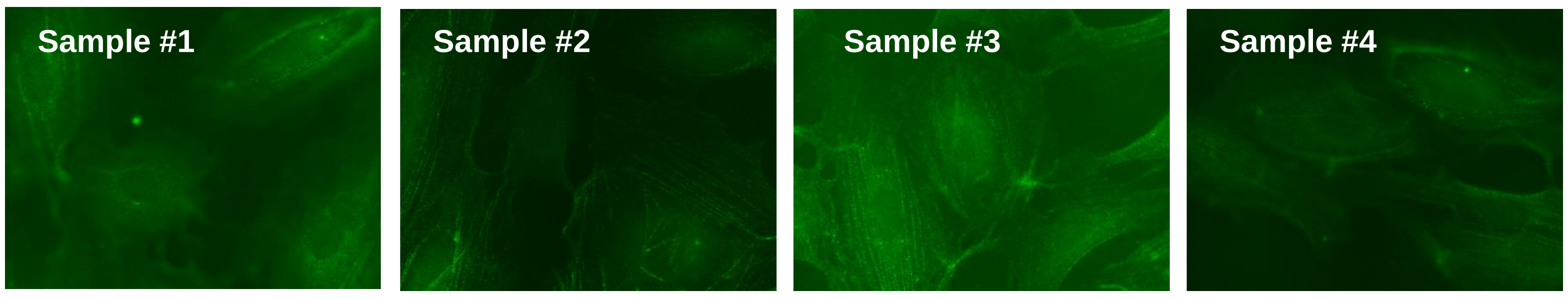

***Pg* OMVs (60 min)**

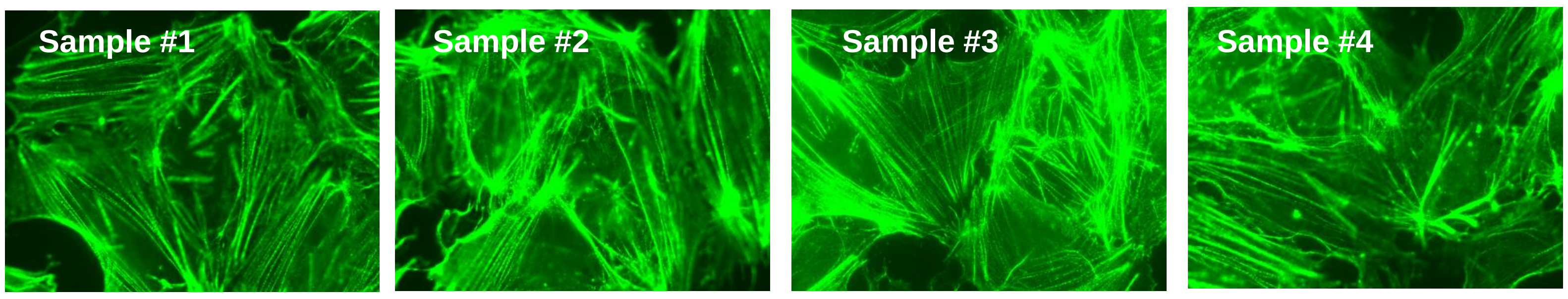

Fluorescence intensity

Group	Sample	Green (F-actin)	Mean
None	#1	26291	**54255**
	#2	10746	
	#3	175673	
	#4	4309	
Pg OMVs	#1	9391448	**15260259**
	#2	16806419	
	#3	20621179	
	#4	14221989	

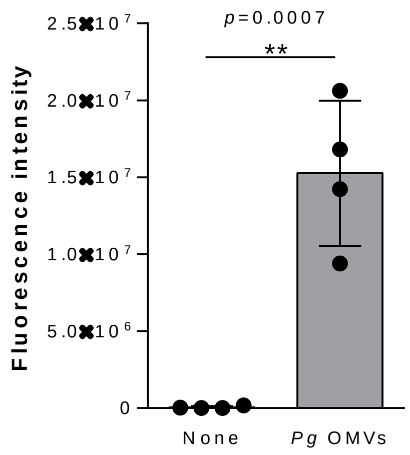

3rd

None

n=4 samples (from different cover glasses) in each group

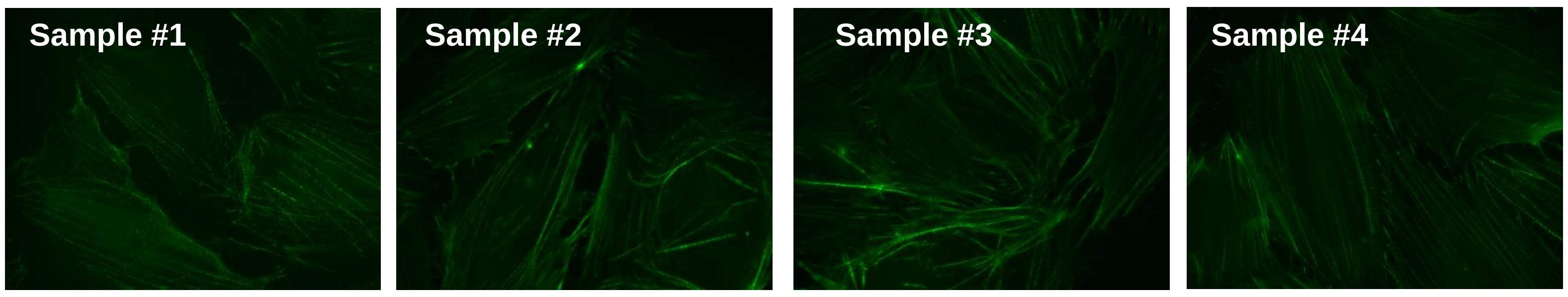

***Pg* OMVs (60 min)**

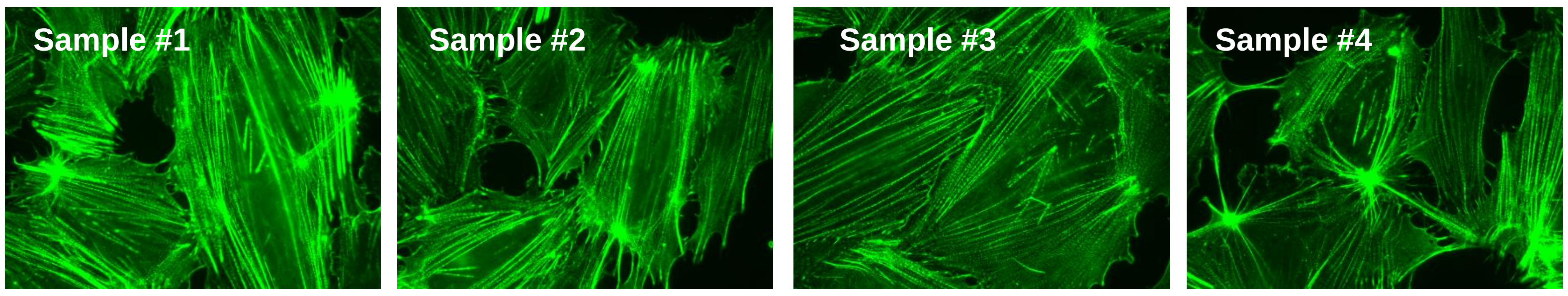

Fluorescence intensity

Group	Sample	Green (F-actin)	Mean
None	#1	382	
	#2	4879	
	#3	92301	
	#4	4987	
Pg OMVs	#1	29036777	
	#2	14540723	
	#3	10757628	
	#4	11322257	

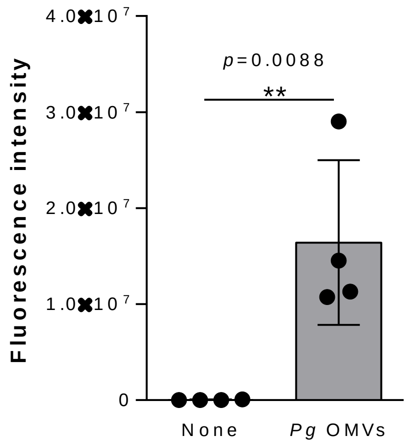

Explanatory material 3: Co-localization of VEc and LAMP-1 (**Figure 4B, i**)

1. View of the fields which were quantified co-localization of VEc and LAMP-1

n=4, samples; *n*=8, field of view in each group

None

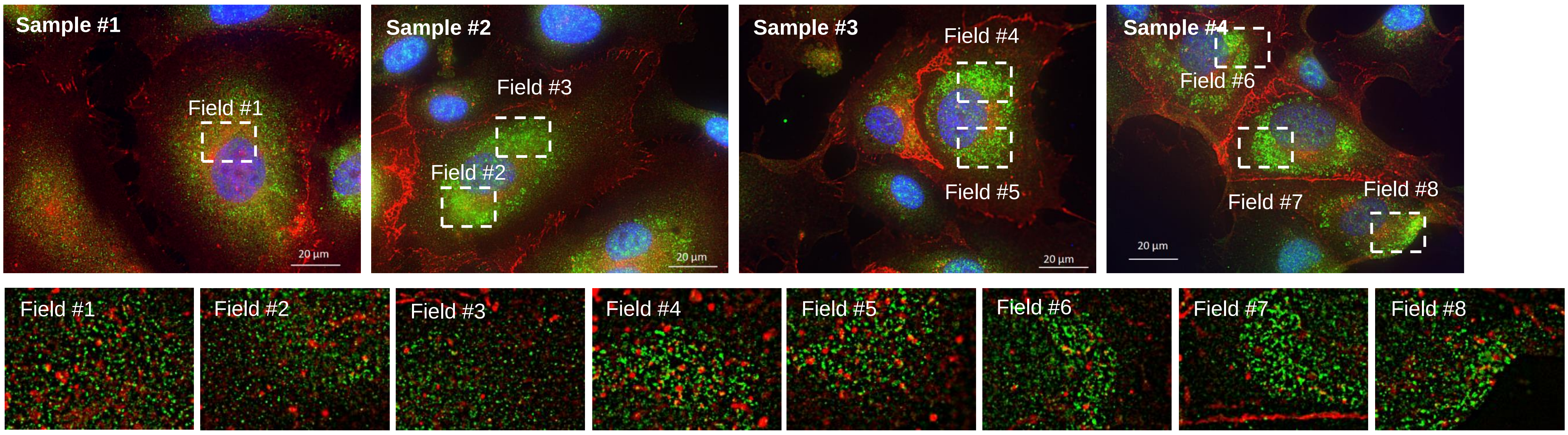

***Pg* OMVs (180 min)**

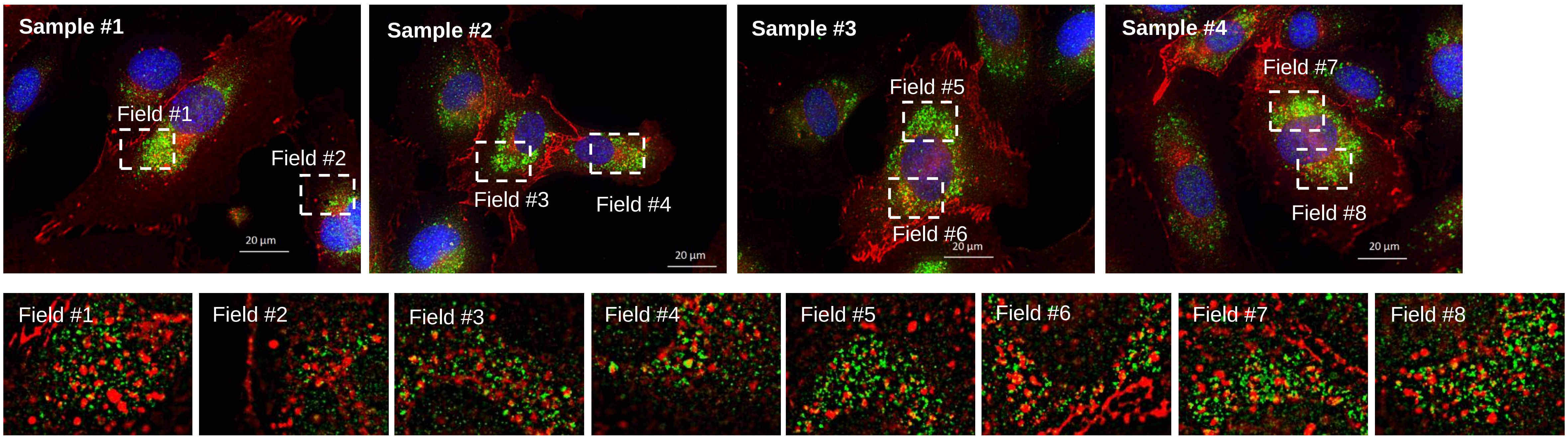

2. Quantification of Yellow (merge) fluorescence and Green fluorescence (LAMP-1) by BZ-X800 Analyzer (Keyence) with Hybrid Cell Count system.

3. Colocalized VEc and LAMP-1 was defined as folloing;

Colocalization coefficients = merge (yellow)/LAMP-1 (green)

4. Result

Fluorescence intensity

Group	Sample	Field	Yellow (Merge)	Green (LAMP-1)	Colocalization efficients (ratio of merge/LAMP-1)	mean
None	#1	#1	10822	386395	**0.0280**	**0.0378**
	#2	#2	8074	285893	**0.0282**	
		#3	2720	212241	**0.0128**	
	#3	#4	26118	381751	**0.0684**	
		#5	19918	299686	**0.0665**	
	#4	#6	9667	286041	**0.0338**	
		#7	8989	367323	**0.0245**	
		#8	12920	319406	**0.0405**	
Pg OMVs	#1	#1	36797	270088	**0.1362**	**0.1309**
		#2	49353	245085	**0.2014**	
	#2	#3	52745	370858	**0.1422**	
		#4	51830	405097	**0.1279**	
	#3	#5	24606	180631	**0.1362**	
		#6	5755	83467	**0.0689**	
	#4	#7	22419	222178	**0.1009**	
		#8	26135	196492	**0.1330**	

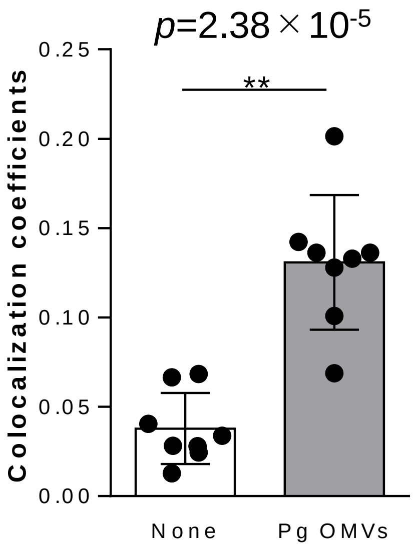

Explanatory material 4: Involvement of Rho A kinase (**Figure 5C**)

n=4 samples (from different cover glasses) in each group

None

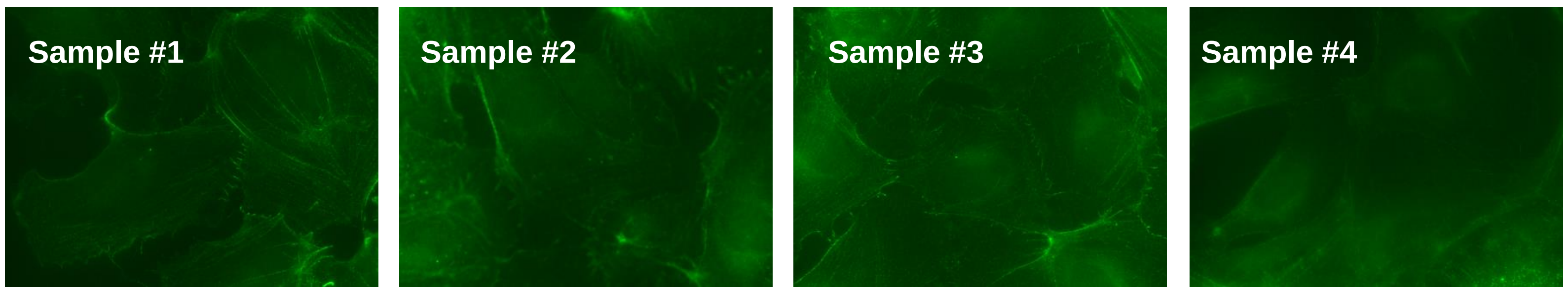

***Pg* OMVs (60 min)**

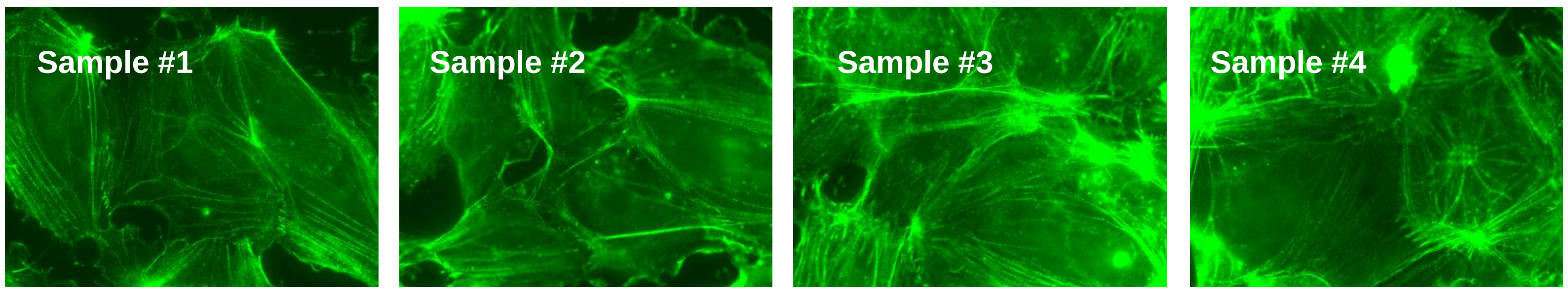

***Pg* OMVs (60 min) + A27632**

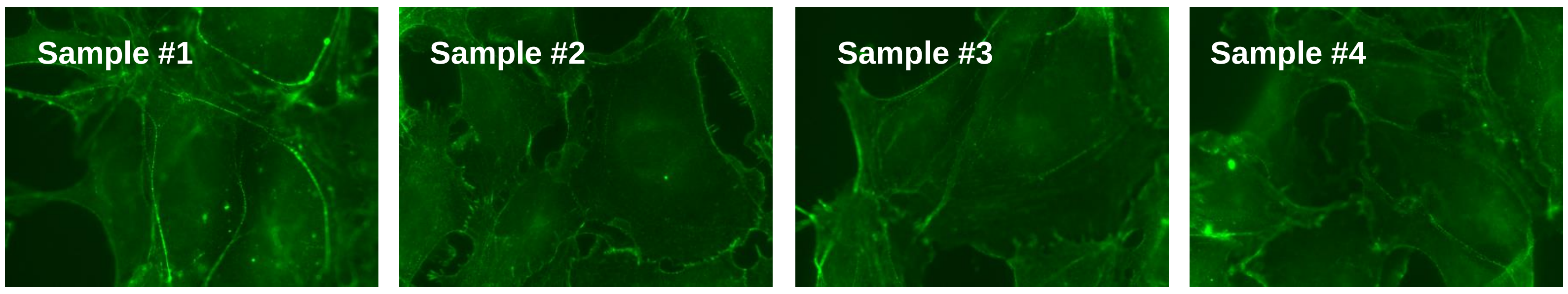

Fluorescence intensity

Group	Sample	Green (F-actin)	mean
None	#1	290350	**997677**
	#2	1182748	
	#3	2012822	
	#4	504787	
Pg OMVs	#1	8815638	**15512632**
	#2	13280142	
	#3	22378053	
	#4	17576696	
Pg OMVs + A27632	#1	2024906	**1315229**
	#2	781962	
	#3	943950	
	#4	1510099	

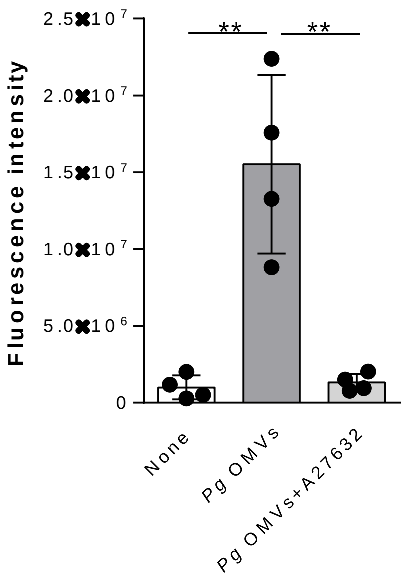

p=0.0026 (None vs *Pg* OMVs)
p=0.0028 (*Pg* OMVs vs *Pg* OMVs+A27632)

Explanatory material 5: Effects of gingipains (**Supplementary Figure 1B**)

n=4 samples (from different cover glasses) in each group

None

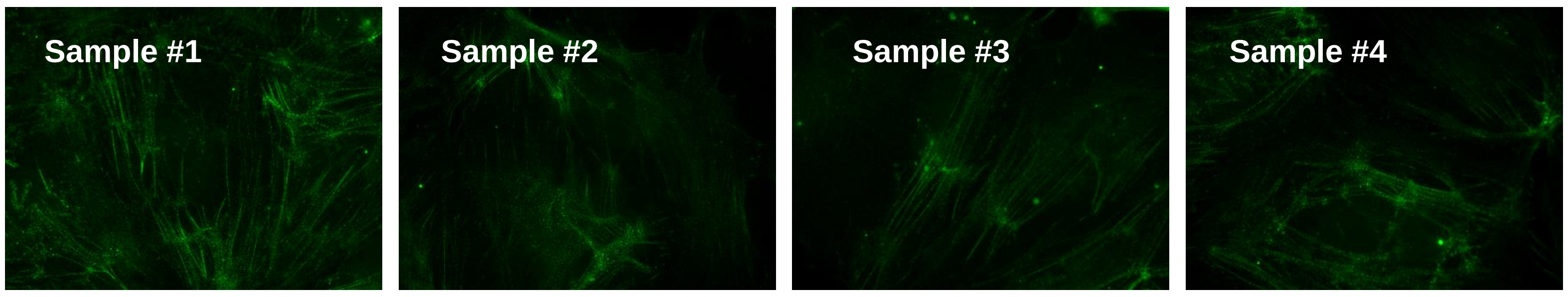

***Pg* OMVs (WT)**

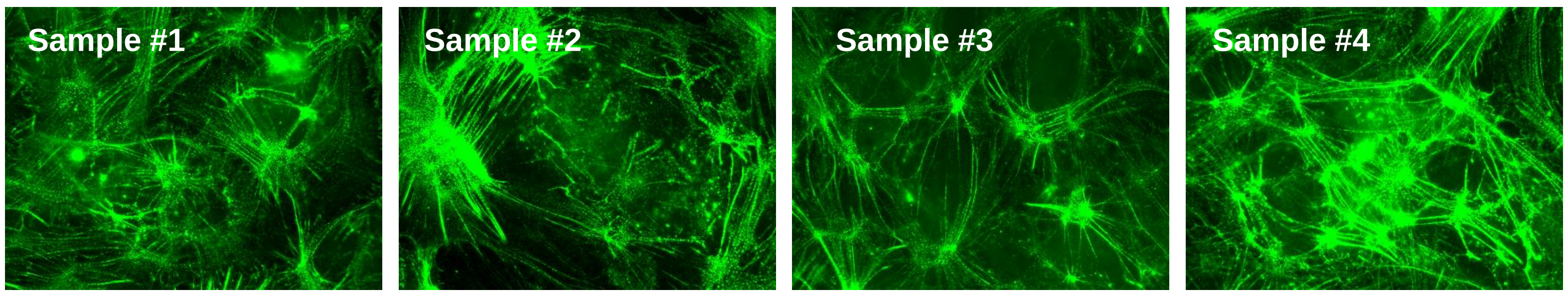

***Pg* OMVs (KDP136)**

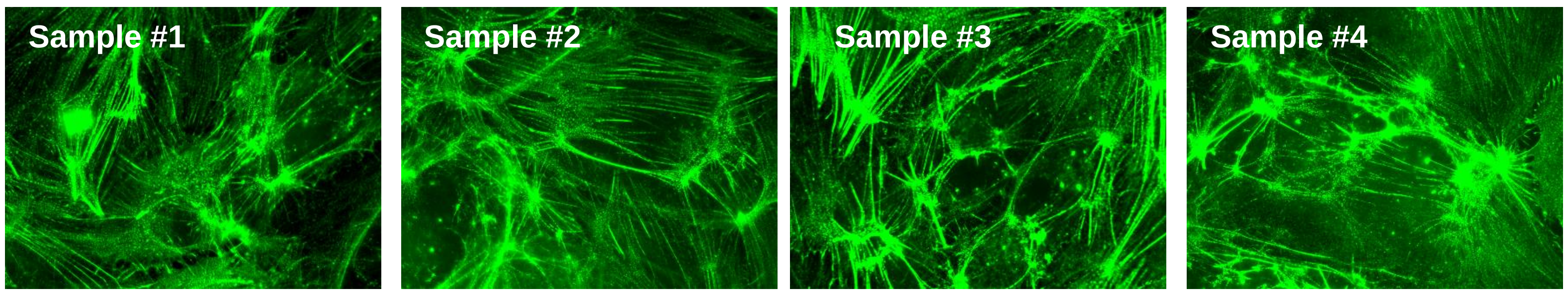

Fluorescence intensity

Group	Sample	Green (F-actin)	mean
None	#1	16842	**13331**
	#2	6256	
	#3	9867	
	#4	20357	
Pg OMVs (WT)	#1	6775376	**11347129**
	#2	12717941	
	#3	4535335	
	#4	21359862	
Pg OMVs (KDP136)	#1	5656953	**12035818**
	#2	10009597	
	#3	17644223	
	#4	14832499	

j

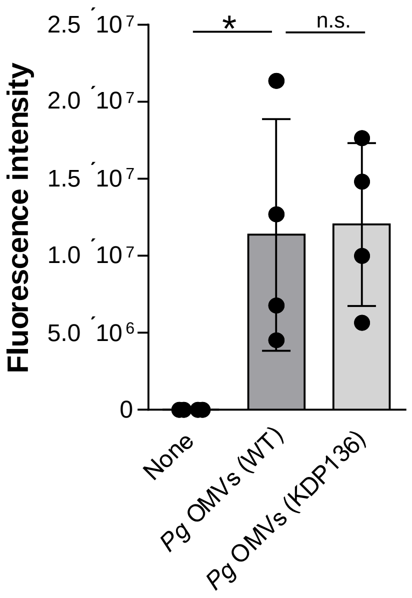

p=0.0024 (None vs *Pg* OMVs WT)
p=0.886 (*Pg* OMVs WT vs *Pg* OMVs KDP136)

editage

Editing Certificate

This document certifies that the paper listed below has been edited to ensure that the language is clear and free of errors. The logical presentation of ideas and the structure of the paper were also checked during the editing process. The edit was performed by professional editors at Editage, a division of Cactus Communications. The intent of the author's message was not altered in any way during the editing process. The quality of the edit has been guaranteed, with the assumption that our suggested changes have been accepted and have not been further altered without the knowledge of our editors.

MANUSCRIPT TITLE

Porphyromonas gingivalis outer membrane vesicles increase vascular permeability by inducing stress fiber formation and VE-cadherin degradation in endothelial cells

AUTHORS

Kaya Yoshida

ISSUED ON

May 21, 2024

JOB CODE

CQFQJ_5_2

THE EDITAGE Promise QUALITY GUARANTEE

Prabh Grewal
Senior Vice President - Editage

editage | helping you get published

Since 2002, Editage has helped over 430,000 authors publish around 1.2 million research papers in scholarly journals across over 1000 disciplines through editorial, translation, transcription, and publication support services. Editage is a brand of Cactus Communications (cactusglobal.com), a science communication and technology company.

CACTUS.

GLOBAL :
+1(833) 979-0061 | request@editage.com

JAPAN :
0120-50-2987 | submissions@editage.com

editage.com | editage.co.kr | editage.jp | editage.cn | editage.com.br | editage.com.tw | editage.de
